# Mechanochemical Upcycling of Polyvinylidene Fluoride: Lewis Acid Induced Generation of Sodium Aluminium Fluorides

**DOI:** 10.1002/cssc.70741

**Published:** 2026-06-22

**Authors:** Minh Bui, Jakob Bölle, Jörg Radnik, Steffen Weidner, Luise Sander, Mike Ahrens, Kerstin Scheurell, Kannan Balasubramanian, Franziska Emmerling, Thomas Braun

**Affiliations:** ^1^ Department of Chemistry Humboldt‐Universität zu Berlin Berlin Germany; ^2^ Federal Institute for Materials Research and Testing (BAM) Berlin Germany

**Keywords:** ball milling, Lewis acid, mechanochemistry, polyvinylidene fluoride, sodium aluminium fluorides

## Abstract

A sustainable mechanochemical process for the generation of sodium aluminium fluorides by conversion of polyvinylidene fluoride (PVDF) waste on using ball milling in the presence of a Lewis acid was developed. The generated fluorides can be key materials for the aluminium production process. The Lewis acid AlCl_3_ initiates dehydrofluorination steps at PVDF, releasing HF for further fluorination of both NaCl and AlCl_3_ to yield chiolite under ball milling conditions. Further calcination of chiolite generates cryolite with an overall yield of 62% with respect to AlCl_3_. The procedure avoids the use of solvents and minimises energy consumption. The identity and phase purity of the products was confirmed by XRD, NMR, IR, and Raman analyses. It was also demonstrated that powdered PVDF, real‐life PVDF membrane waste or PVDF extracted from Li‐ion batteries can be upcycled into industrially relevant fluoride materials. The presented method offers a sustainable approach for resource recovery and environmental remediation.

## Graphical abstract

A solvent‐free mechanochemical process to upcycle PVDF waste into sodium aluminium fluorides, which are essential for aluminium production, was developed. By ball milling PVDF with AlCl_3_ and NaCl, the polymer degrades into chiolite and graphitic materials. Subsequent calcination then yields cryolite. This sustainable route turns persistent PFAS waste into industrial resources, bridging environmental remediation and circular chemistry.

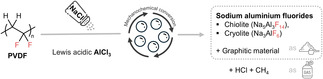

## 1 Introduction

Sodium aluminium fluorides are raw materials in aluminium manufacturing. In particular, their crucial role as flux agents in the Hall–Héroult process demonstrates their importance for industry [1]. Cryolite (Na_3_AlF_6_) is essential and acts as the primary flux for dissolving Al_2_O_3_ in the electrolysis process. The presence of cryolite in an Al_2_O_3_ bath significantly lowers the melting temperature from approximately 2000°C to 1000°C by forming an eutectic system, thereby reducing the immense energy consumption required for the aluminium production [2]. Beyond cryolite, other sodium aluminium fluorides such as chiolite (Na_5_Al_3_F_14_) could be observed in the molten electrolyte of the Hall‐Héroult process [3]. Common protocols to synthesise cryolite involve a two‐step process. Initially, H_2_SiF_6_—a byproduct from the phosphoric acid production, because of the presence of HF—reacts with Al(OH)_3_ and NH_3_ to form (NH_4_)_3_AlF_6_. Subsequently, the intermediate is converted into the Na_3_AlF_6_ by reaction with NaCl [4]. Due to their industrial importance and given that natural mineral deposits are virtually depleted, there is a strong need for sustainable routes to access sodium aluminium fluoride compounds [5].

Polyvinylidene fluoride (‐[CH_2_CF_2_]_n_‐, PVDF) belongs to the class of the per‐ and polyfluoroalkyl substances (PFAS) and is used for instance as a binder in Li‐ion batteries or as a coating for electronic and chemical processing equipment [6, 7]. Its exceptional chemical robustness is based on the strong C–F bond [8, 9, 10]. However, this chemical robustness allows PVDF to persist and accumulate in ecosystems [11, 12, 13]. Various strategies have been developed to address this problem. Morita et al. demonstrated the separation of PVDF from poly(ethylene terephthalate) in photovoltaic backsheets via NaOH hydrolysis [14]. They improved this process by using tetrabutylammonium bromide and CaCl_2_ to recover F^−^ as CaF_2_ [15]. While the CaF_2_ produced via this route can in principle serve as a feedstock for synthetic cryolite, the conversion requires multiple additional steps, which include the generation of HF from reacting H_2_SO_4_ with CaF_2_. Furthermore, Sarkar et al. have shown the recovery and reuse of PVDF binders from cathode films in Li‐ion batteries by delamination of the film and subsequent extraction of PVDF by using various solvents including THF (tetrahydrofuran), DMF (dimethylformamide), NMP (*N*‐methylpyrrolidone) and their respective mixtures [16]. Importantly, the groups of Gouverneur and Shibata demonstrated the mechanochemical degradation of PFAS, including PVDF in the presence of either K_3_PO_4_ or KO*t*Bu to form KF [17, 18].

It has recently also been shown that the mechanochemical degradation of PVDF can be achieved by using Lewis acidic AlCl_3_ to give AlF_3_ and graphitic products that are functionalised to a low extent by fluoride and chloride substituents [19]. The conversion involves a dehydrofluorination mechanism, which generates HF. On the other hand, Scholz et al*.* showed that cryolite or chiolite can be synthesised via the mechanochemical conversion of NaF and AlF_3_ [20, 21]. Interestingly, product formation depends on the molar ratio of the reactants and the modification of the AlF_3_. In particular, the use of amorphous AlF_3_ leads to chiolite, while the use of crystalline α‐AlF_3_ resulted in the formation of cryolite.

**SCHEME 1:**
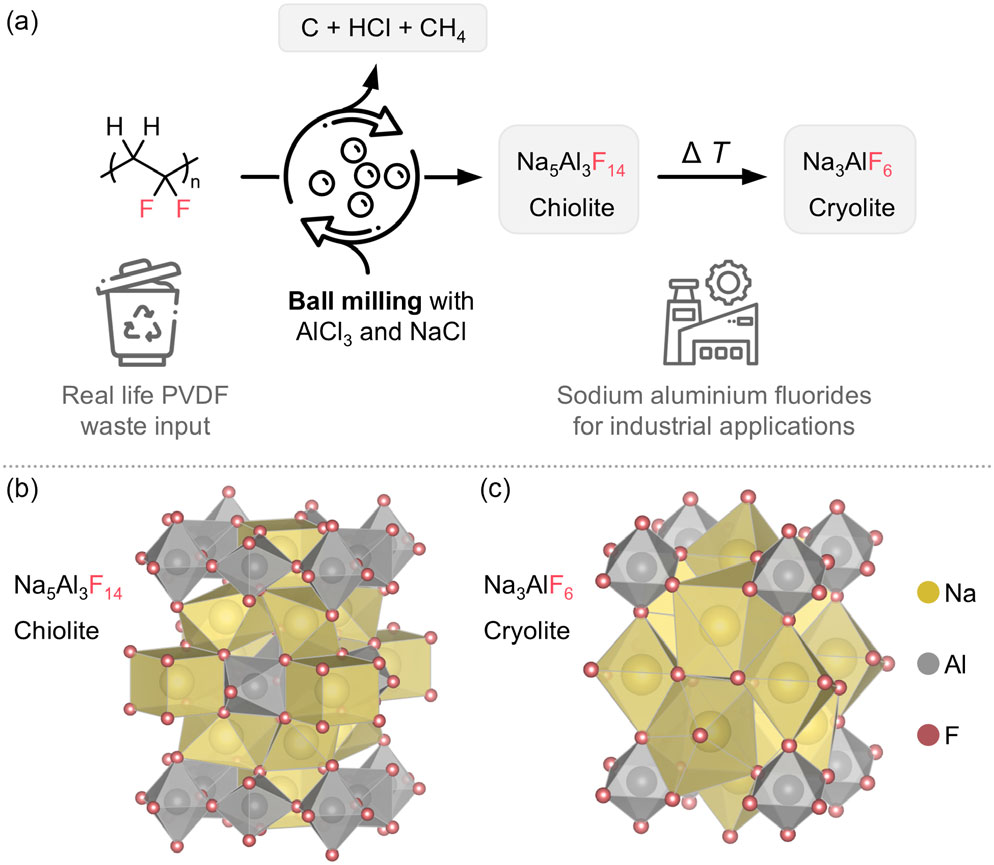
(a) Mechanochemical synthesis of sodium aluminium fluorides by ball milling PVDF, NaCl and Lewis acidic AlCl_3_. Crystal structures of (b) chiolite [22] and (c) cryolite [23].

The present work demonstrates a method that addresses both industrial needs and environmental concerns by converting PVDF. We report on the mechanochemical conversion of PVDF into chiolite (Na_5_Al_3_F_14_), graphitic products and HCl by using Lewis acidic AlCl_3_ and NaCl as co‐milling agents. The obtained chiolite can then be converted to cryolite by calcination (Scheme 1).

## 2 Results and Discussion

Mechanochemical conversion of PVDF into sodium aluminium fluorides was performed in a planetary mill. NaCl, AlCl_3_ and PVDF were placed in an Ar glovebox into a ZrO_2_ jar equipped with gassing lids and ZrO_2_ balls. After milling, the gaseous products (HCl, CH_4_ and H_2_) were vacuum transferred at ‐196°C from the jars into JYoung NMR tubes, which were filled with C_6_D_6_. The solutions were used for characterisation. The obtained solids after milling appeared as fine black powders made of spherical particles with a diameter of ca. 100–300 nm (elemental mapping using STEM and EDX, SI Figure 18), which consist of AlF_3_, Na_5_Al_3_F_14_ as well as fluorinated and chlorinated graphite. Different milling times and various molar ratios of the starting compounds were tested for the synthesis of sodium aluminium fluorides which are listed in Table 1.

**TABLE 1:** Milling parameters for the synthesis of chiolite and the composition of the powder determined by Rietveld refinement.

Entry	Molar ratio of substrates			Milling time in h	Composition (wt.%) of the milled powder mixture^a^			
	NaCl	AlCl_3_	PVDF^b^		Na_5_Al_3_F_14_	NaCl	α–AlF_3_	am.^c^
1	3	1	3	4	17	24	3	56
2^d^	3	1	3	4	11	23	4	62
3^e^	3	1	1.5	4	49	13	1	37
4^d^^,^ e	3	1	1.5	4	40	25	2	33
5	1	2	3	7	4	7	14	75
6	1.6	1	2.3	7	16	13	19	52
7	1.5	1	3	4	52	1	3	44
8	1.5	1	3	7	48	2	3	47
9^f^	1.5	1	3	4	44	3	5	48
10^g^	1.5	1	6	4	32	3	0	65

a calculated by Rietveld refinement from powder XRD, CaCO_3_ as internal standard.

b equivalents of CH_2_CF_2_ entities.

c am.: amorphous compounds, which could be PVDF, amorphous AlF_3_ and graphitic entities.

d milling was conducted in an air atmosphere, before milling the jar was exposed to air.

e NaF was used instead of NaCl.

f PVDF membrane was used instead of powdered PVDF.

g PVDF was extracted from a Li‐ion battery.

### 2.1 Powder X‐Ray Diffraction

**FIGURE 1:**
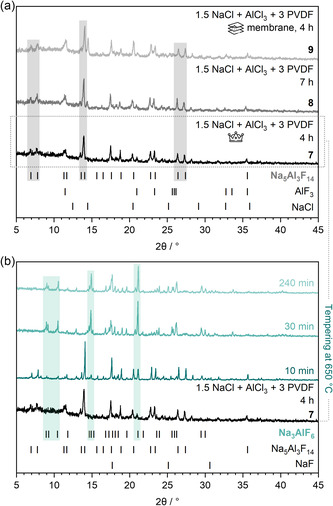
(a) Selected PXRD (Mo Kα source *λ* = 0.7107 Å) diffractograms from milling approaches of entry 7, 8 and 9 from Table 1. The XRD patterns of the samples with CaCO_3_ as internal standard are listed in the SI. The reference reflections for Na_5_Al_3_F_14_, AlF_3_ and NaCl are depicted as black lines. Intense Na_5_Al_3_F_14_ reflections are highlighted in grey. (b) Monitoring the structural change of entry 7 from tempering at 650°C in air for various time by PXRD analysis. Selected reference reflections for Na_5_Al_3_F_14_, Na_3_AlF_6_ and NaF are depicted as black lines. Intense Na_3_AlF_6_ reflections are highlighted in green.

**FIGURE 2:**
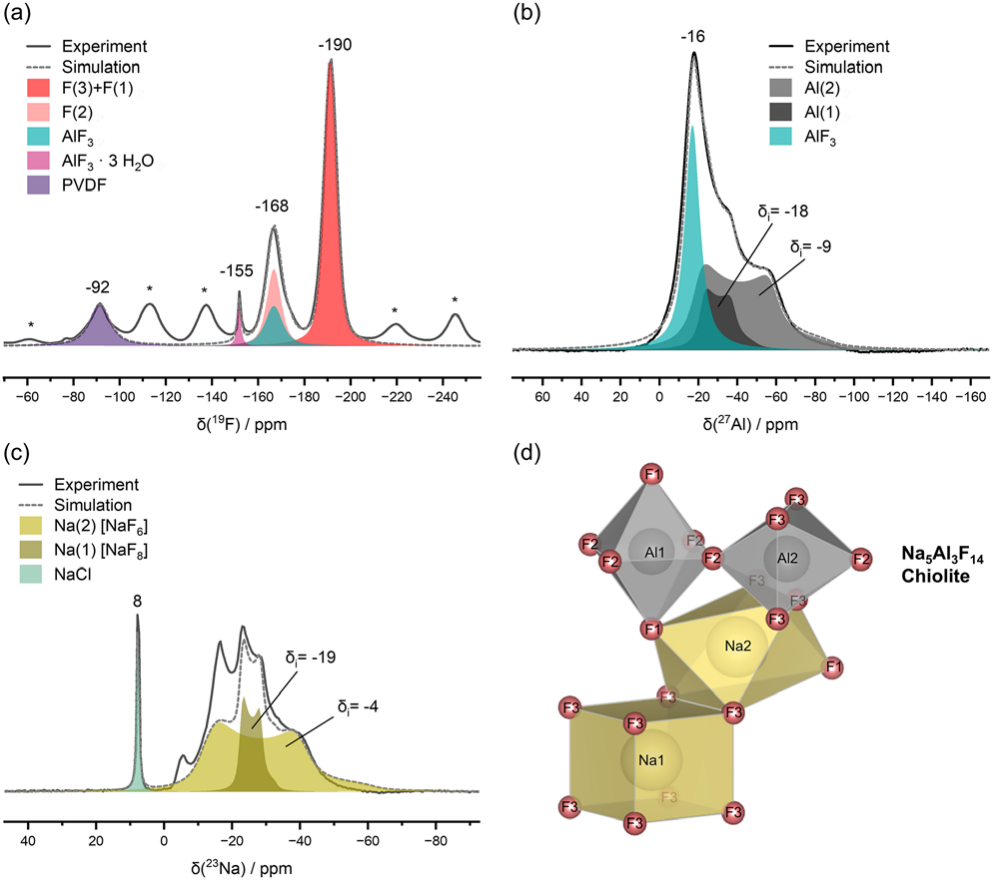
(a) ^19^F MAS NMR, (b) ^27^Al MAS NMR and (c) ^23^Na MAS NMR (for all: ˜v_rot_ = 20 kHz) spectra of the milling reaction entry 8 from Table 1. Isotropic chemical shifts are given as δ_i_. Asterisks (*) are representing spinning sidebands. Simulation of the spectra were conducted with DMFIT [24]. (d) Selected polyhedra for displaying the different sites in the crystal structure of chiolite [22].

For all obtained powders, PXRD diffractograms were acquired (Figure 1) and the composition of the milled mixtures were investigated by Rietveld refinement. Interestingly, when milling with a 3:1:3 ratio of NaCl, AlCl_3_ and PVDF (i.e. CH_2_CF_2_ moiety as 1 eq. PVDF) (Table 1, entry 1), PXRD analysis revealed that the resulting powder consisted of 17 wt.% of Na_5_Al_3_F_14_, 24 wt.% of NaCl, and 3 wt.% of AlF_3_ for the crystalline phases (Figure 1a). The generation of Na_3_AlF_6_ was not observed despite the preset ratio of starting compounds, likely because the milling process preferentially produces amorphous AlF_3_ as evidenced by MAS NMR spectroscopy and PXRD (see below, Figure 2). Accordingly, Scholz et al*.* observed that milling NaF with amorphous AlF_3_ resulted in the formation of chiolite instead of cryolite [20, 21]. Note that amorphous graphitic materials cannot be observed in the PXRD pattern. Testing the reaction with the same molar ratio but in an air atmosphere instead of an argon atmosphere (Table 1, entry 2) resulted in a decrease of the amount of Na_5_Al_3_F_14_ by 6 wt.%. This could be due to the blockage of the Lewis acidic sites of AlCl_3_ by the moisture. Using NaF instead of NaCl in the milling reaction (Table 1, entry 3) led to an increased conversion of PVDF to yield Na_5_Al_3_F_14_, probably because there is no need to generate NaF from NaCl by reaction with HF. For entry 6 in Table 1 a ratio of NaCl, AlCl_3_ and PVDF of 1.6:1:2.3 was tested to achieve a stochiometric conversion into Na_5_Al_3_F_14_. However, the crystalline products contained only 16 wt.% of chiolite. The main product was α‐AlF_3_ with 19 wt.%, and 13 wt.% of the starting NaCl remained unreacted.

When the molar ratio of AlCl_3_ to PVDF was 1:3 and the amount of NaCl was lowered by half compared (1.5:1:3 for NaCl:AlCl_3_:PVDF) to entry 1, a high conversion into Na_5_Al_3_F_14_ was found (Table 1, entry 8) with a ratio of Na_5_Al_3_F_14_ of 49 wt.% and α‐AlF_3_ of 4 wt.%. Minor intensities for the reflections of NaCl in the PXRD diffractogram were observed revealing a content of 1 wt.% NaCl. Note that for the generation of sodium aluminium fluorides, the conversion of NaCl to NaF by HF is required. This HF is generated in situ via a Lewis acidic interaction between PVDF and AlCl_3_. With a 2:3 ratio of AlCl_3_ to PVDF, most of the released HF tends to react predominantly with AlCl_3_ rather than with NaCl. This is likely due to the preferred Al–F bond (675 kJ/mol) formation compared to the Na–F interaction (477 kJ/mol) [25]. Consequently, to enhance the desired NaF formation, it is crucial to reduce the amount of AlCl_3_ relative to PVDF. In the context of waste streams, a real life PVDF membrane sample was then tested and the PXRD analysis indeed showed generation of Na_5_Al_3_F_14_, with a content of 44 wt.% (Table 1, entry 9). Furthermore, PVDF was extracted from a Li‐ion battery and investigated towards mechanochemical conversion into chiolite. Since this PVDF material was not obtained in pure form (see SI), an excess of this polymer was used. The resulting product composition consists of 32 wt.% chiolite, 3 wt.% NaCl, and 65 wt.% amorphous material (entry 10, Table 1). These results are promising for the conversion of an everyday PVDF material.

After identifying the milling conditions in entry 7 to be optimal milling due to its high chiolite content and minimal residual NaCl, the obtained mixture was then subjected to a calcination step to convert chiolite into cryolite and to assess the scalability of the overall process. Sample 7 was then chosen to be tempered at 650°C in air to monitor its reaction from chiolite to cryolite (Table 2): 3 Na_5_Al_3_F_14_ → 5 Na_3_AlF_6_ + 4 AlF_3_. This temperature was selected because it provides a balance between the solid‐state reaction of Na_5_Al_3_F_14_ to yield Na_3_AlF_6_ and to minimise any conversion of the metal fluorides into the metal oxides. Because the tempering was performed in air, the generated AlF_3_ likely reacted with atmospheric O_2_ to form amorphous Al_2_O_3_. This is supported by MAS NMR spectroscopic investigations (see below). After 30 min of tempering, PXRD data depicted in Figure 1b showed a decrease in the reflection intensities for chiolite and the simultaneous emergence of reflections corresponding to cryolite (Na_3_AlF_6_). A prolonged tempering (240 min at 650°C in air) of the initial dark powder mixture resulted in formation of a white powder and a mass loss of nearly 50 wt.% (SI, Figure 21), due to the oxidation of the graphitic material to CO_2_. For the experiment in entry 7, after calcination at 650°C for 4 h in air Na_3_AlF_6_ was produces in 62% yield based on the used AlCl_3_ was determined (SI, Figure 12). TGA and DSC are discussed in the SI.

**TABLE 2:** Composition of the crystalline phases of the calcinated (at 650°C) powder mixture of entry 7 from Table 1.

Entry^a^	Ratio (wt.%) of the crystalline phases from the powder during the calcination process^b^					
	Na_3_AlF_6_	Na_5_Al_3_F_14_	NaCl	AlF_3_	NaF	Al_2_O_3_
7	—	90	—	10	—	—
7_10	27	70	—	—	1	2
7_30	92	3	1	—	—	4
7_60	93	2	—	—	1	4
7_120	92	1	1	—	2	4
7_180	87	2	1	—	6	5
7_240	87	1	1	—	5	6

a time in min in the tempering process are given after the underline.

b calculated by Rietveld refinement from powder XRD.

### 2.2 MAS NMR

Figure 2a shows the ^19^F MAS NMR spectrum of the sample after milling experiment outlined in entry 8 (Table 1), which displays five signals. The spectrum confirms the presence of Na_5_Al_3_F_14_ with signals for its F(3) and F(1) fluorine sites at −190 ppm and a distinct, very broad signal for the F(2) site at −165 ppm [26]. An underlying signal at −168 ppm is attributed to AlF_3_ [27]. Furthermore, a resonance for AlF_3_ · 3 H_2_O was found at −155 ppm [28], and −(CF_2_CH_2_)_n_− moieties from residual PVDF were identified by a signal at −92 ppm. In the ^27^Al MAS NMR spectrum (Figure 2b) three signals were observed. A signal at −16 ppm can be assigned to AlF_3_, while two signals at *δ*_i_ = −18 ppm and *δ*_i_ = −9 ppm with quadrupolar splitting are due to the Al(1) and Al(2) sites of chiolite [21, 26]. The ^23^Na MAS NMR spectrum in Figure 2c exhibits a signal at −8 ppm, which is indicative of residual NaCl. A very broad signal is also present, which can be deconvoluted to two signals with quadrupolar splitting, characteristic of the Na(1) and Na(2) sites of chiolite [21, 26]. Spectral simulations suggest the presence of additional, unidentified sodium species. The ^13^C MAS NMR spectrum is discussed in the SI.

**FIGURE 3:**
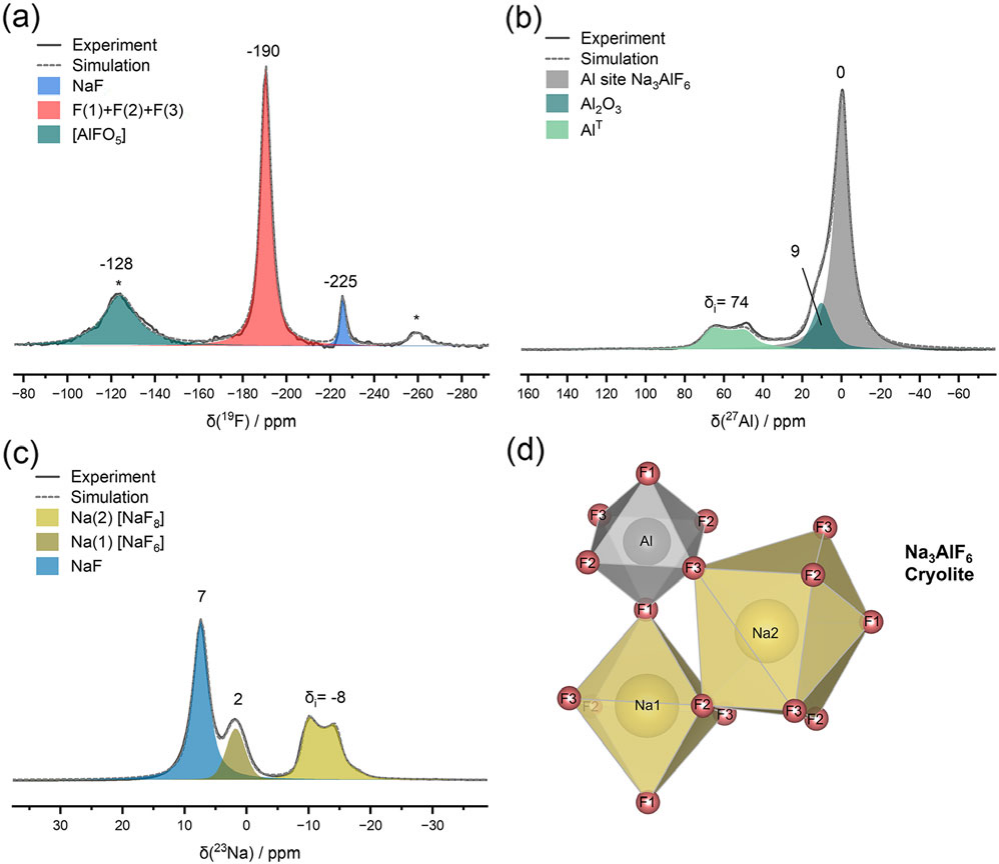
(a) ^19^F MAS NMR, (b) ^27^Al MAS NMR and (c) ^23^Na MAS NMR (for all: ˜v_rot_ = 20 kHz) spectra of the milling reaction given in entry 7_240 from Table 2 after heating in air at 650°C for 4 h. Isotropic chemical shifts are given as δ_i_. Asterisks (*) represent spinning sidebands. Al^T^ stands for tetrahedral coordinated aluminium. Simulations of the spectra were conducted with DMFIT [24]. (d) Selected polyhedra for displaying the different sites from the crystal structure of cryolite [23].

MAS NMR spectra of the sample 7_240 heated for 240 min in air at 650°C (Table 2) are displayed in Figure 3. The ^19^F MAS NMR spectrum (Figure 3a) shows three distinct signals. A sharp signal at −225 ppm is assigned to NaF [29]. The major signal centred at −190 ppm corresponds to the three chemically distinct fluorine sites of cryolite (Figure 3d) [21]. The third signal is very broad, with an underlying spinning sideband at −128 ppm. This signal is characteristic for [AlFO_5_] octahedra [30, 31, 32]. The ^27^Al MAS NMR spectrum of sample 7_240 reveals the presence of three distinct aluminium species (Figure 3b). The dominant resonance is observed at 0 ppm and is characteristic for the Al sites in Na_3_AlF_6_ [21]. A shoulder at 9 ppm indicates the presence of aluminium oxide [33, 34], which is, as mentioned above, likely formed due to the thermal treatment of the sodium aluminium fluoride mixture in an air atmosphere. A third signal, appearing at *δ*_i_ = 74 ppm with a quadrupolar splitting (SI Table 3), is consistent with tetrahedrally coordinated aluminium centres [33, 34]. In the ^23^Na MAS NMR spectrum (Figure 3c) two signals corresponding to the Na sites in Na_3_AlF_6_ are observed. The signal at 2 ppm is assigned to the Na(1) site. The signal for the Na(2) site with a quadrupolar splitting appears at *δ*_i_ = −8 ppm. In addition, a signal for NaF at 7 ppm is observed [35].

### 2.3 ATR and Raman Spectroscopy

**FIGURE 4:**
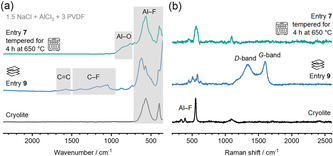
(a) ATR‐IR and (b) Raman (recorded at 532 nm) spectra of entry 7_240 (Table 2), entry 9 (Table 1) and neat cryolite as reference.

ATR IR spectroscopic analysis (Figure 4a) of the powder mixture of entry 9 using a PVDF membrane (Table 1) reveals characteristic Al−F vibrational modes for Na_5_Al_3_F_14_ at 620 cm^−1^ with a shoulder at 570 cm^−1^ [36, 37]. The C−F vibrational bands at 1400–1000 cm^−1^ originate from residual PVDF [38, 39]. Additionally, a notable C = C vibrational mode is detected at 1590 cm^−1^ [40]. Upon heating a sample in air at 650°C for 240 min, the IR spectrum for entry 7_240 changes significantly. The C−F bands disappear while the shape of the IR spectrum becomes comparable with the IR spectrum of neat cryolite (Na_3_AlF_6_). In addition, a broad Al−O band was observed at 900 cm^−1^ [41], suggesting the above mentioned formation of Al_2_O_3_ during the heating process.

Raman spectra (Figure 4b) of both samples of entry 7_240 (Table 2), entry 9 (Table 1) reveal distinct *D*‐band and *G*‐bands, which are characteristic for graphitic materials. The Raman spectrum of sample 9 shows the *D‐* and *G‐*band at 1338 and 1601 cm^−1^, respectively. The *D*‐band indicates the presence of structural disorder or defects, such as folds, cracks, and edge discontinuities. In contrast, the *G*‐band corresponds to the in‐plane stretching vibration of sp^2^‐C bonds, indicating the presence of a graphitic structure [42, 43]. Additionally, several Al−F bands between 450–650 cm^−1^ can be attributed to chiolite [44]. After heating the sample in air at 650°C for 240 min, the Raman spectrum undergoes significant changes. As mentioned, this temperature was chosen to achieve a balance between the solid‐state reaction from Na_5_Al_3_F_14_ to Na_3_AlF_6_ and minimising the conversion to the metal oxides. A broad band emerged at 552 cm^−1^, which is attributed to an Al–F mode. This confirms the successful formation to cryolite, consistent with the presence of pure cryolite [45].

### 2.4 XPS Spectroscopy

**FIGURE 5:**
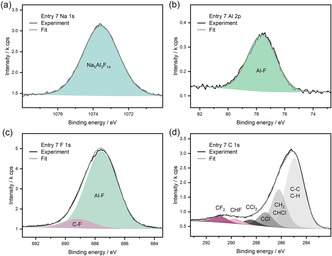
(a) Na 1s, (b) F 1s, (c) Al 2p, and (d) C 1s high resolution XPS spectra of the milling sample 7 from Table 1.

XPS spectra of sample 7 (Table 1) are displayed in Figure 5. For the ‘chiolite’ rich powder mixture the Na 1s XPS spectrum (Figure 5a) shows a signal at 1073.6 eV. This binding energy is notably high when compared to reference materials in standard databases, such as for Na_3_AlF_6_ at 1071.8 [46], NaF at 1072.7 [47], or NaCl at 1071.8 eV [48]. A direct match with these sodium compounds was not observed. Note also that there are no published XPS data for Na_5_Al_3_F_14_ to date. The Al 2p XPS spectrum exhibits one signal at 77.6 eV assigned to Al–F. In the F 1s XPS spectrum, two signals at 689.1 for C−F moieties and 687.6 eV for Al−F species were detected. Comparing the C 1s data with data of KF‐black obtained by the Shibata group show no presence of C–O entities, but a greater structural complexity of the carbonaceous material was revealed after ball‐milling of PVDF in the presence of AlCl_3_ and NaCl [18]. The C 1s XPS spectrum shows signals for CF_2_ at 290.8, CHF at 289.6, CCl_2_ at 288.4, CCl at 287.3, CH_2_ or CHCl at 286.2 and C−C or C−H moieties at 284.8 eV. Analysis of the Cl 2p spectrum (SI Figure 30) provides further support for the presence of C−Cl moieties on the graphitic material. A clear detection of a distinct sp^2^ C peak for graphitic entities at approximately 284.8 eV was not possible, because the expected π to π* transition peak [49] at 291–293 eV cannot be identified due the obscured CF_2_ peak in this region [19].

### 2.5 Mass Balance of the PVDF Conversion Under Optimised Reaction Conditions

**FIGURE 6:**
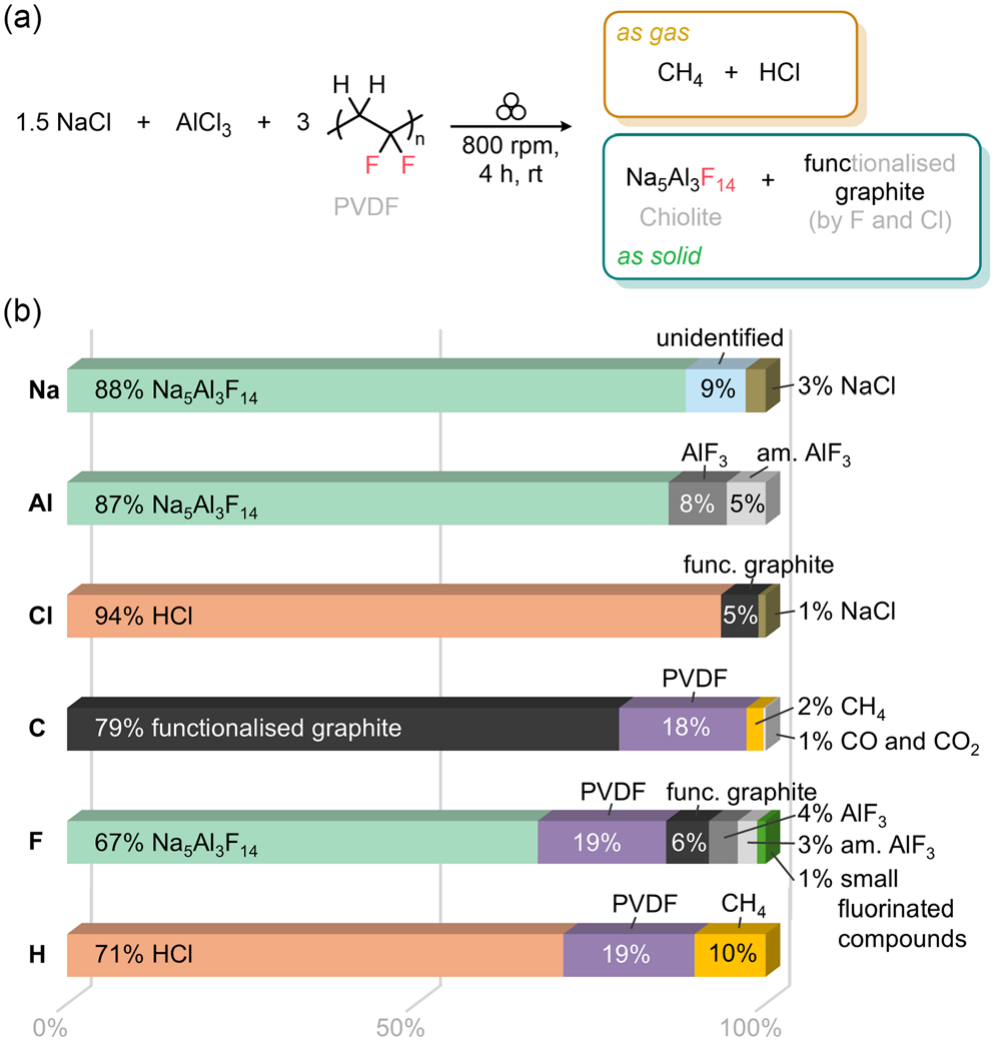
(a) Optimised reaction condition for the mechanochemical synthesis of chiolite (entry 7, Table 1). (b) Mass balance of the elements for the products and unreacted starting compounds based on OFCEAS, PXRD, XPS and MAS NMR spectroscopy.

Based on the high PVDF conversion observed in milling experiment 7 (entry 7, Table 1, Figure 6a), an estimation of an elemental mass balance was derived to track the distribution of the elements in the reaction by combining data from MAS NMR spectroscopy, PXRD, XPS, and OFCEAS (Figure 6b). The initial milling jar loading contained 450 mg of reactants. Following the milling process, 278 mg of a black powder was recovered, while the remaining 172 mg of mass loss was assigned to gaseous products. The amorphous phase contains 2 wt.% AlF_3_ and 10 wt.% PVDF as determined by subsequent MAS NMR spectroscopic studies. For the carbonaceous part of the amorphous fraction (32 wt.% of the overall product) was recovered as graphite, which still contains C−Cl and C−F bonds. However, Figure 6b shows the mass balance for each element in mol%, for comparison. The Rietveld data from Table 1 of the residue indicate that 88 mol% of the Na and 87 mol% of the Al from NaCl and AlCl_3_ are incorporated into the chiolite phase. Generated gases during milling were vacuum transferred into a JYoung NMR tube containing C_6_D_6_. Optical feedback cavity enhanced absorption spectroscopy (OFCEAS) revealed a gaseous composition of 96.6 mol% HCl, 1.5 mol% CH_4_, 1.3 mol% CO_2_ and 0.6 mol% CO (SI Figure 31). The CO_2_ and CO content is likely attributable to air ingress. Furthermore, 94 mol% of the chlorine content originating from both NaCl and AlCl_3_ is released as HCl gas. While the fluorine content from the PVDF is mainly converted into solids (Na_5_Al_3_F_14_, unreacted PVDF, functionalised graphite, AlF_3_ and amorphous AlF_3_), a minor content of small fluorinated compounds could also be detected (approximately 1% of the green‐house gases CF_3_H, CF_3_CH_3_, SI Figure 34). Previous studies of the mechanochemical degradation of the binary AlCl_3_/PVDF system demonstrated that the use of a 2:3 stoichiometric ratio of AlCl_3_ to PVDF (CF_2_CH_2_ as 1 equivalent) resulted in no detectable small fluorinated molecules [19].

### 2.6 Proposed Reaction Mechanism

**SCHEME 2:**
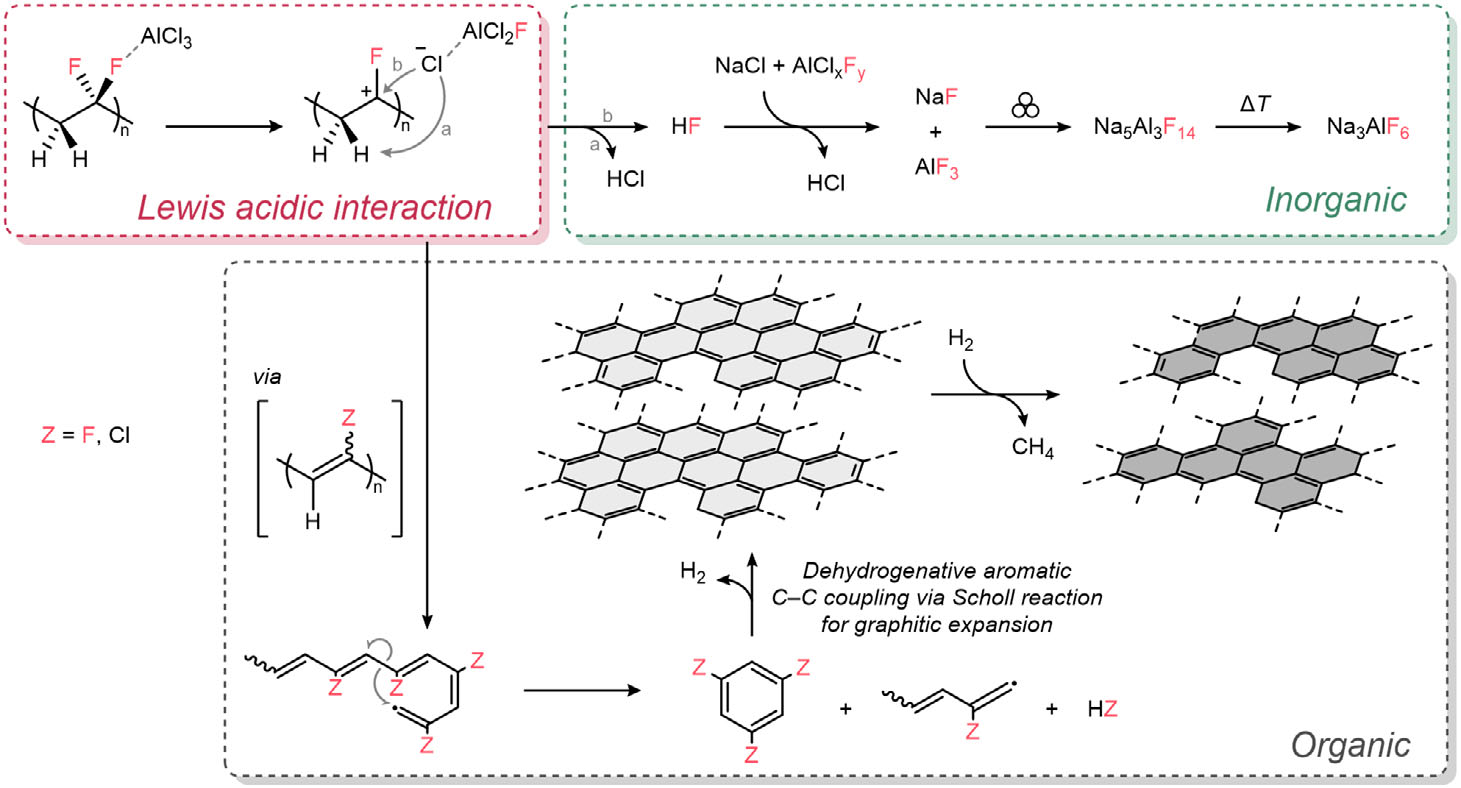
Proposed reaction mechanism for the mechanochemical synthesis of sodium aluminium fluorides using AlCl_3_, NaCl, and PVDF.

Built upon the previous results, the following reaction pathway is proposed (Scheme 2) [19]. The initial step for the synthesis of sodium aluminium fluorides is presumably a Lewis acidic interaction of AlCl_3_ with a C–F bond of the –(CF_2_CH_2_)_n_− moiety to give a carbenium‐like species and [AlCl_3_F]^–^. Two reaction pathways are then possible. Thus, [AlCl_3_F]^–^ might abstract a proton at the carbenium species to release HCl (dehydrochlorination, a). Alternatively, the carbenium intermediate could be chlorinated by the [AlCl_3_F]^–^ anion. This step is then followed by Lewis‐acid‐supported HF elimination (dehydrofluorination, b) [50, 51, 52], which produces a partially chlorinated olefinic species. The dehydrohalogenated polyolefinic species then undergo C–C coupling reactions to form halogenated aromatic rings, releasing more HF and HCl (the latter was confirmed by optical feedback cavity enhanced absorption spectroscopy (OFCEAS) SI Figure 31). Finally, a Lewis‐acid‐mediated Scholl reaction generates H_2_ (^1^H NMR spectrum, SI Figure 32), which leads to the formation of fluorine and chlorine functionalised graphitic entities (Figure 5d) [53, 54]. These graphitic entities can be detected in LDI‐TOF analysis of the milled powders (SI Figure 25, 26). Model reactions involving the ball milling of 2‐fluoronapthalene and 2‐chloronapthalene in the presence of a catalytical amount of AlCl_3_ resulted in the formation of graphitic bands as confirmed in the Raman spectrum (SI Figure 29). The released H_2_ from the Scholl reaction creates a reductive environment that activates the graphitic material, which in turn generates CH_4_ [55, 56]. The generated HF finally fluorinates the Na and Al salts to yield the corresponding metal fluorides. Test reactions have shown that NaCl cannot react itself mechanochemically with PVDF or AlF_3_ to form NaF (SI Figure 2). Once NaF and AlF_3_ are formed, a mechanochemical conversion into Na_5_Al_3_F_14_ can occur [20, 21].

### 2.7 Scalability and Process Sustainability

The viability of the mechanochemical route is supported by the high conversion into chiolite. Elemental mass balance under optimised conditions (entry, 7, Table 1, Figure 6) confirms that 87–88% of the Na and Al from the precursors are incorporated into sodium aluminium fluorides. This provides a direct pathway for fluorine circularity, as these products are valuable intermediates for the Hall‐Héroult process. Although the reaction involves a significant gas‐phase fraction, the stream consists almost entirely of HCl (97%) with no detectable HF. In a commercial setting, this concentrated HCl stream can in principle be valorised as aqueous HCl or CaCl_2_ using standard scrubbing techniques, which avoids the large volumes of diluted aqueous waste typically generated by wet‐chemical PFAS treatments [57]. The process for PVDF depletion was further validated by treating PVDF, which was extracted from spent lithium‐ion batteries (Table 1, entry 10). While chiolite formation was achieved, a higher amount of this PVDF extract was required to reach comparable conversion like using neat PVDF. This can be attributed to the lower purity of the battery‐sourced material and possible structural alterations or degradation of the polymer during its operational lifetime [58, 59]. The data indicate that the developed process is compatible with non‐pure PVDF and can accommodate the chemical variations of real‐world waste. In terms of safety and reactor stability, the thermodynamic preference for Al−F and Na−F bond formation ensures that fluorine is rapidly trapped in the solid phase as AlF_3_ and NaF, which effectively prevents the release of HF gas. Corrosive HCl in the gas phase would be managed using established engineering solutions, such as industrial mills or continuous mechanochemical reactors lined with corrosion‐resistant nickel and ceramic alloys [60]. Operating under slightly negative pressure with continuous gas extraction is common practice in the fluorochemical and aluminium‐smelting industries [61], so the proposed route does not present an unusual safety challenge. The sustainability of the process is further supported by the nature of the carbonaceous byproduct. Rather than generating mobile perfluoroalkyl substances or diffuse microplastics, the reaction yields a condensed, largely aromatic graphitic solid. With 10–15% halogen (F, Cl) functionalisation (confirmed by XPS), this material is recovered as a stable solid that significantly reduces environmental mobility compared to the original PVDF. Confining the carbon into a collectable solid phase represents a meaningful improvement over traditional landfilling or incineration, where fluorine recovery is typically absent [62].

## 3 Conclusion

This work establishes a solvent‐free mechanochemical platform that can convert PVDF (polyvinylidene fluoride, −[CH_2_CF_2_]_n_−) waste directly into sodium aluminium fluorides. PVDF belongs to the class of per‐ and polyfluorinated substances, which are persistent in the environment. The mechanochemical process integrates the degradation of the polymer, the recovery of resources, and the generation of key components of the Hall–Héroult flux in a single, scalable operation. Fine control over milling conditions and stoichiometry enables the high‐yield formation of chiolite from neat PVDF and from real life PVDF waste in form of membranes or extracted from Li‐ion batteries. While not yet fully valorised for the battery‐derived PVDF, this solid fraction offers potential for further upcycling into functional carbons or energy recovery via controlled combustion. The obtained chiolite is then transformed quantitatively into cryolite via calcination, as corroborated by complementary diffraction and spectroscopic analyses. As well as providing an efficient solid‐state synthesis of industrially relevant fluorides, this strategy introduces a general approach to the environmentally friendly utilisation of persistent fluoropolymers and provides a model for future PFAS upcycling technologies.

## Funding

This study was supported by Deutsche Forschungsgemeinschaft (ID 387284271, SFB 1349).

## Conflicts of Interest

The authors declare no conflicts of interest.

## Supporting information

The authors have cited additional references within the Supporting Information [63, 64, 65, 66, 67, 68, 69, 70, 71, 72].
