# Supplementary material for: Mechanochemical Upcycling of Polyvinylidene Fluoride: Lewis Acid Induced Generation of Sodium Aluminium Fluorides
Source: ChemSusChem. 2026 Jun 22;19(12):e70741. doi: 10.1002/cssc.70741 (PMC13285255; doi:10.1002/cssc.70741)
Supplement: Supplementary file 1 — The authors have cited additional references within the Supporting Information [63, 64, 65, 66, 67, 68, 69, 70, 71, 72]. [file CSSC-19-e70741-s001.pdf]

Supplementary Information

Mechanochemical Upcycling of Polyvinylidene Fluoride: Lewis Acid Induced Generation of Sodium Aluminium Fluorides

Minh Bui,[a] Jakob Bölle,[a] Jörg Radnik,[b] Steffen Weidner,[b] Luise Sander,[b] Mike Ahrens,[a] Kerstin Scheurell,[a] Kannan Balasubramanian,[a] Franziska Emmerling*,[a,b] and Thomas Braun*;[a]

[a] Department of Chemistry, Humboldt Universität zu Berlin, Brook-Taylor Str. 2, 12489 Berlin, Germany

[b] Federal Institute for Materials Research and Testing, Richard-Willstätter Str. 11, 12489 Berlin, Germany

[c] School of Analytical Sciences Adlershof (SALSA) & IRIS Adlershof, Albert-Einstein Str. 11, 12489 Berlin, Germany

Table of Contents

General information and materials 2

Safety precautions 2

PVDF extraction from a Li-ion battery 3

Mechanochemistry 3

Optimal milling conditions 4

Tempering approaches 5

Powder X-ray reflection diffraction (PXRD) 6

Scanning / transmission electron microscopy 15

Solid-state MAS NMR spectroscopy 16

Thermogravimetric analysis (TGA) and differential scanning calometry (DSC) 19

Laser-desorption ionisation (LDI) time of flight (TOF) Mass spectrometry ... 21

Infrared spectroscopy 23

Raman spectroscopy 24

X-ray photoelectron spectroscopy 24
Analytics for the gaseous content 27
Optical feedback cavity enhanced absorption spectroscopy (OFCEAS) 27
Liquid NMR spectroscopy 28

General information and materials

All samples were prepared in an MBraun glovebox filled with argon or in JYoung NMR tubes using conventional Schlenk techniques. The materials were sourced as follows: NaCl (99.5 %) from Carl Roth, which was dried under reduced pressure before use; anhydrous $AlCl_3$ (99.99 %) from ABCR; and polyvinylidene fluoride (PVDF) as powder (M_w = 600.000) or membrane (ROTI®Fluoro, 0.2 µm pore size) from BLDpharm and Carl Roth, respectively. Benzene-d_6 from Eurisotop was used as received. Reference compounds, including cryolite and α-AlF_3 from Sigma Aldrich and 2-chloronaphthalene and 2-fluoronaphthalene from TCI and Apollo Scientific, were all used without further purification. Dimethylformamide (DMF, 99.8 %) and dimethyl carbonate (DMC, 99 %) were purchased from Carl Roth and Thermo Fisher Scientific and used as received.

Safety precautions

During the ball-milling procedure the intermediate HF generation might occur, but is reacts immediately with $AlCl_3$ or NaCl to produce stable metal fluorides. However, safety measures are mandatory. Appropriate safety precautions must be taken when using HF, which is a highly toxic and irritant compound. Severe burns can be caused if HF comes in contact with the skin. Working with HF must include wearing proper personal protective equipment, working in a fume hood and having calcium gluconate gel immediately available. Additionally, we recommend jars and balls made of ZrO_2 and the usage of fluorinated O-rings.

PVDF extraction from a Li-ion battery

An MJ1 18650 Li-ion battery from LG Energy Solution was opened carefully by removing the plastic sleeve followed by dismantling the metal case with a suitable cutter and pliers (SI Figure 1). Extreme care was taken to avoid damaging the separator during the opening process of the battery case. The individual layers (cathode, anode and separator) were then unrolled and isolated from one another. Battery components were rinsed in DMC to remove the electrolytes. 20 g of the cathode was then cut into small pieces and stirred in DMF for 12 h at 80 °C. The resulting black suspension was passed through an alumina column to obtain a yellowish filtrate. After removing DMF from the filtrate under reduced pressure, 283 mg of an off-white yellowish film could be obtained. While ^{19}F NMR and IR spectroscopic analysis (SI Figures 37 and 27) identified the material as PVDF, the data suggest that the extraction process did not yield a completely pure product. Subsequently, the film was used for the mechanochemical conversion of PVDF to chiolite by co-milling with NaCl and $AlCl_3$.

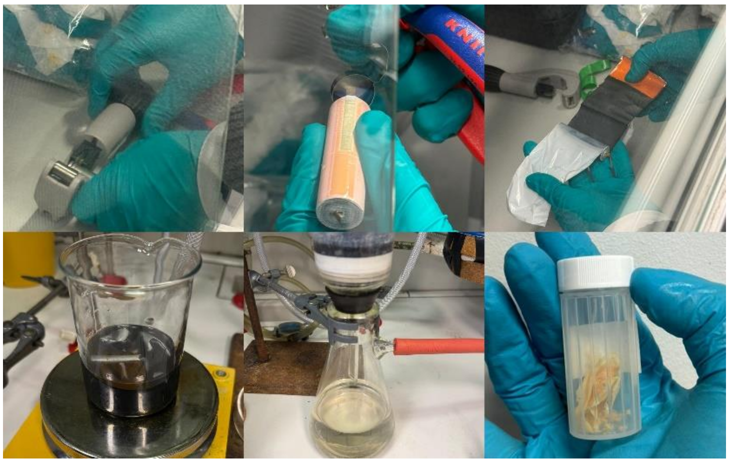

SI Figure 1. Sequence of opening a Li-ion battery for PVDF recovery.

Mechanochemistry

Planetary milling was conducted in a Fritsch Premium Line 7 using 45 mL ZrO_2 jars equipped with a Swagelok gassing lid, a Viton® O-ring and five ZrO_2 balls (each 2.5 g, 10 mm diameter) with a rotational speed of 800 rpm.

a) Anhydrous NaCl, anhydrous $AlCl_3$ and PVDF as a powder, as a membrane or extracted from a Li-ion battery were placed into jars with a total mass of 450 mg. The

molar ratio of the starting compounds can be found in the SI in Table 1. The commercially available PVDF membrane (ROTI®Fluoro pore size 0.2 µm) was milled for 4x30 min (2 h) before usage.

b) Mixtures of 1 eq. 2-chloronapthalene, 3 eq. 2-fluoronapthalene with and without 10 mol% $AlCl_3$ (in total 450 mg) were milled for 4 h. In absence of $AlCl_3$, no reaction was observed. However, when 10 mol% $AlCl_3$ was added a dark grey powder was obtained. This product was washed with 5 x 3 mL of isopropanol and analysed using Raman spectroscopy.

c) To better understand how NaCl is fluorinated during the reaction, two additional experiments were performed. α-AlF_3 was milled with NaCl, and PVDF with NaCl for 4 h. The products were analysed using PXRD.

Optimal milling conditions

Process optimisation was conducted by varying the milling temperature, rotational speed, and duration. Higher mechanical energy was found to be critical. While milling at 800 rpm for at least 4 h yielded a homogeneous, fine black powder, reducing the speed (400 rpm) or duration (2 h) resulted in polymer agglomeration and poor conversion to chiolite. Furthermore, pre-tempering the milling jars at 100 °C did not provide an improvement in conversion efficiency. Quantification of the phase composition via PXRD was precluded for samples processed at lower rotational speeds or shorter milling durations, as the material exhibited significant agglomeration and macroscopic inhomogeneity. The presence of residual PVDF flakes further indicated incomplete physical breakdown and prevented the preparation of a representative powder for diffraction analysis.

SI Table 1. Milling parameters for the synthesis of sodium aluminium fluorides.

Entry	Molar ratio of substrates			Milling time in h	Composition (weight%) of the milled powder mixture [a]			
	NaCl	$AlCl_3$	PVDF [b]		$Na_5Al_3F_{14}$ Chiolite	NaCl	α–AlF_3	am. [c]
1	3	1	3	4	17	24	3	56
2 [d]	3	1	3	4	11	23	4	62
3 [e]	3	1	1.5	4	49	13	1	37
4 [d, e]	3	1	1.5	4	40	25	2	33
5	1	2	3	7	4	7	14	75
6	1.6	1	2.3	7	16	13	19	52
7	1.5	1	3	4	52	1	3	44
8	1.5	1	3	7	48	2	3	47
9 [f]	1.5	1	3	4	44	3	5	48
10 [g]	1.5	1	6	4	32	3	0	65
11 [h]	1.5	1	3	2	29	10	9	52
12	1.5	1	3	2	not determined			
13 [i]	1.5	1	3	4	not determined			

[a] calculated by Rietveld refinement from powder XRD, $CaCO_3$ as internal standard. [b] equivalents of CH_2CF_2 entities. [c] am.: amorphous compounds, which could be PVDF; amorphous AlF_3 and graphitic entities. [d] milling was conducted in an air atmosphere, before milling the jar was exposed to air. [e] NaF was used instead of NaCl. [f] PVDF membrane was used instead of powdered PVDF. [g] PVDF was extracted from a Li-ion battery. Note that the PVDF is not pure. [h] Milling jar was pre-heated at 100 °C. [i] the rotational speed was reduced to 400 rpm.

Tempering approaches

Calcination of the samples were conducted in air in a Thermolyne Muffle Furnance by Thermo Fisher Scientific using alumina trays.

a) In a tray sample 7 from SI Table 1 was placed in a preheated furnace at 650 °C for 240 min. During the heating process at defined time points PXRD diffractograms were

recorded from the calcinated powder at 10, 30, 60, 120 and 240 min. After 240 min of heating a white powder could be obtained.

b) 3 eq. NaCl, 1 eq. $AlCl_3$ and 3 eq. PVDF (450 mg in total) are placed in a tray and tempered at 750 °C for 4 h as a test reaction. The calcinated obtained white powder mixture was analysed by PXRD.

Powder X-ray reflection diffraction (PXRD)

Powder X-ray diffraction data were collected on a STOE Stadi MP diffractometer equipped with a Dectris Mythen 1 K linear silicon strip detector and Ge(111)double-crystal monochromator (Mo Kα radiation with λ = 0.7107 Å) in a transmission geometry. The measurements were performed with a step size of 0.5 ° (2θ) with an integration time of 150 s per step over a range of 5-50 ° (2θ). Some samples were measured on a D8 Discover diffractometer (Bruker AXS, Karlsruhe, Germany) operated in transmission geometry (Cu Kα radiation with λ = 1.5406 Å) and equipped with a Lynxeye detector. Rietveld refinement analysis was conducted by using the software Profex.[1] $CaCO_3$ was used as internal standard.

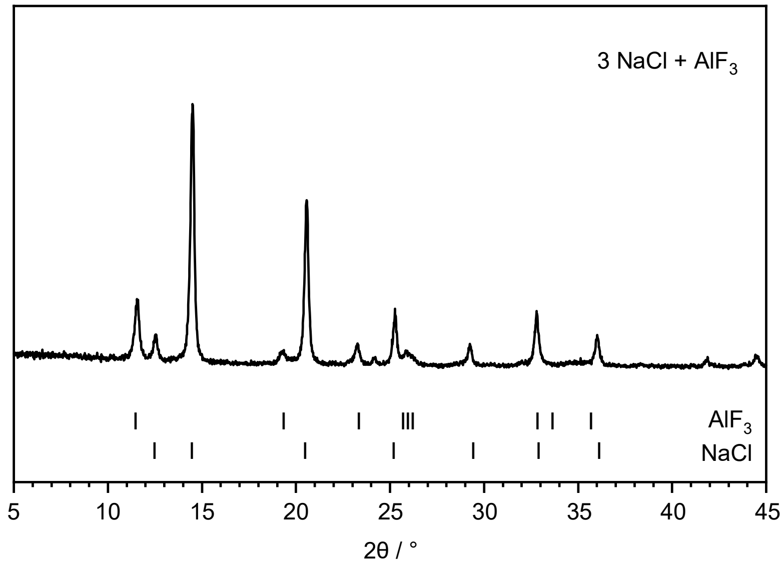

SI Figure 2. PXRD diffractogram (Mo Kα source λ = 0.7107 Å) from milling 3 eq. NaCl and 1 eq. α-AlF_3. The reference reflections for α-AlF_3 and NaCl are depicted in black.

After the mechanochemical treatment of 3 eq. NaCl and 1 eq. AlF_3 a white powder mixture was obtained. No conversion to NaF and $AlCl_3$ were observed according to the PXRD analysis. The PXRD diffractogram in SI Figure 1 only depicts the reflections for

the starting material, indicating that AlF_3 cannot be used for the fluorination of NaCl to yield NaF under mechanochemically conditions.

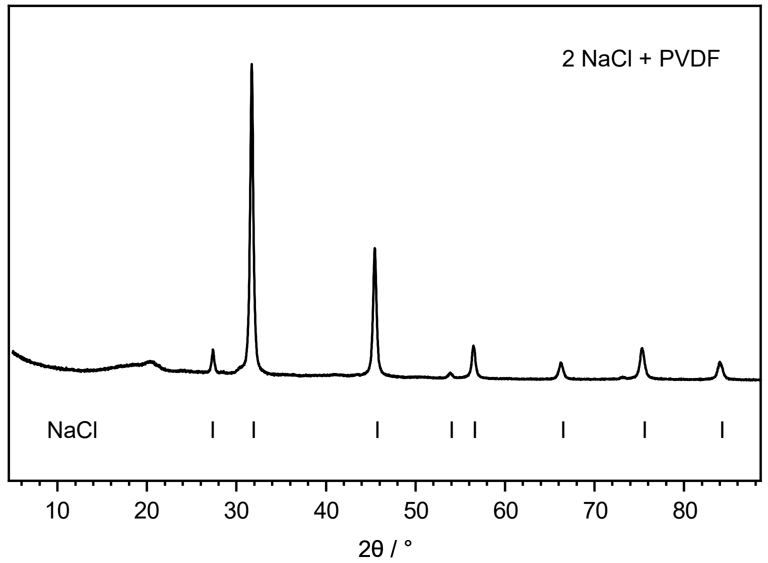

SI Figure 3. PXRD diffractogram (Cu Kα source λ = 1.5406 Å) from milling 2 eq. NaCl and 1 eq. PVDF (CF_2CH_2 as 1 eq.). The reflections of NaCl are depicted in black.

The mechanochemical reaction of 2 eq. NaCl and 1 eq. PVDF (CF_2CH_2 as 1 eq.) leads to a white powder mixture consisting of flat white flakes. The reaction did not lead to the formation of NaF, indicating that PVDF is not suitable for the fluorination of NaCl under these conditions. Only reflections for the starting compound NaCl could be detected in the PXRD diffractogram (SI Figure 3).

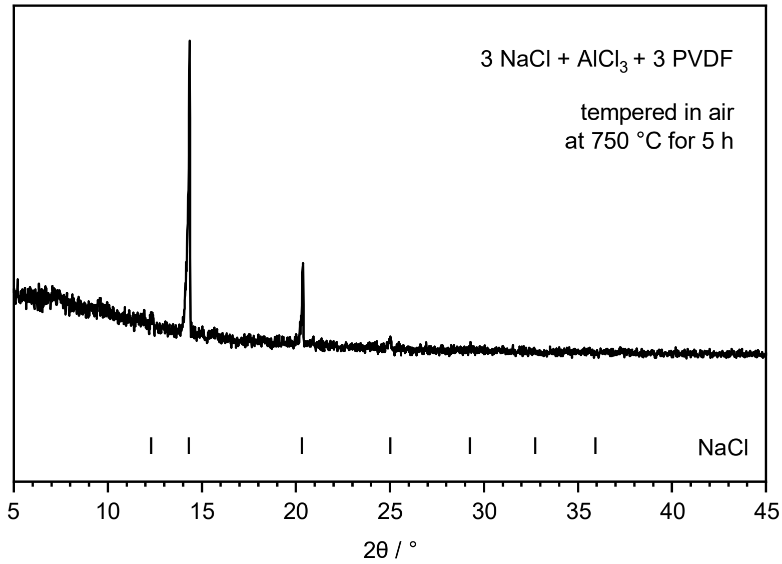

SI Figure 4. PXRD diffractogram (Mo Kα source λ = 0.7107 Å) of the calcinated mixture containing 3 eq. NaCl, 1 eq. $AlCl_3$, and 3 eq. PVDF in air at 750 °C for 5h. The reflections of NaCl are depicted in black.

After the tempering process of 3 eq. NaCl, 1 eq $AlCl_3$, and 3 eq. PVDF in air at 750 °C for 5 h a white powder mixture could be obtained. The PXRD diffractogram (SI Figure 4) of the calcinated mixture shows only reflections for NaCl. $AlCl_3$ might have reacted with released HF from PVDF during heating [2] to form amorphous AlF_3. Calcination of the starting materials did not lead to the formation of sodium aluminium fluorides.

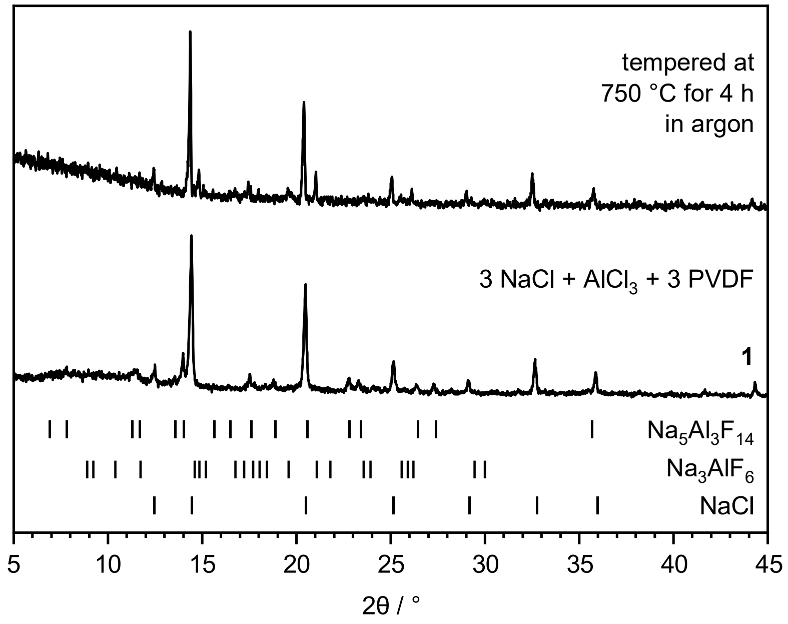

SI Figure 5. PXRD diffractogram (Mo Kα source λ = 0.7107 Å) of milling approach entry 1 alongside that of the calcinated powder at 750 °C for 4 h in argon.

Sample 1 (SI Table 1) was calcinated at 750 °C for 4 h in an argon atmosphere. The corresponding PXRD diffractogram (SI Figure 5) shows the conversion of chiolite ($Na_5Al_3F_{14}$) to cryolite (Na_3AlF_6).

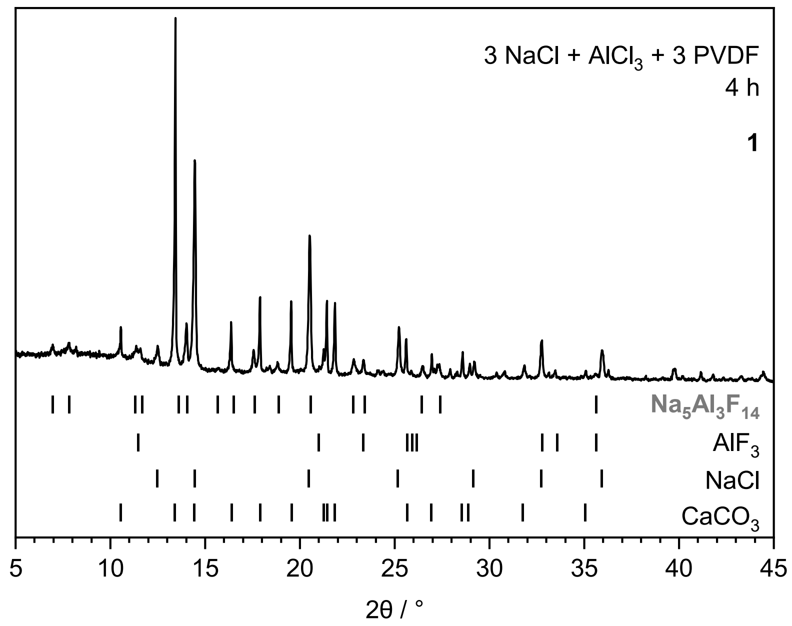

SI Figure 6. PXRD diffractogram (Mo Kα source λ = 0.7107 Å) of milling approach entry 1 with $CaCO_3$ as internal standard.

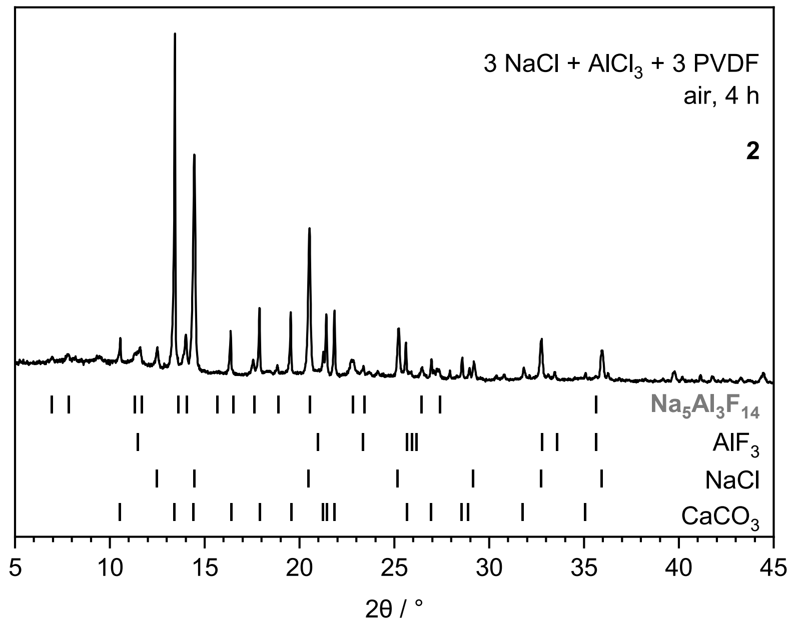

SI Figure 7. PXRD diffractogram (Mo Kα source λ = 0.7107 Å) of milling approach entry 2 with $CaCO_3$ as internal standard.

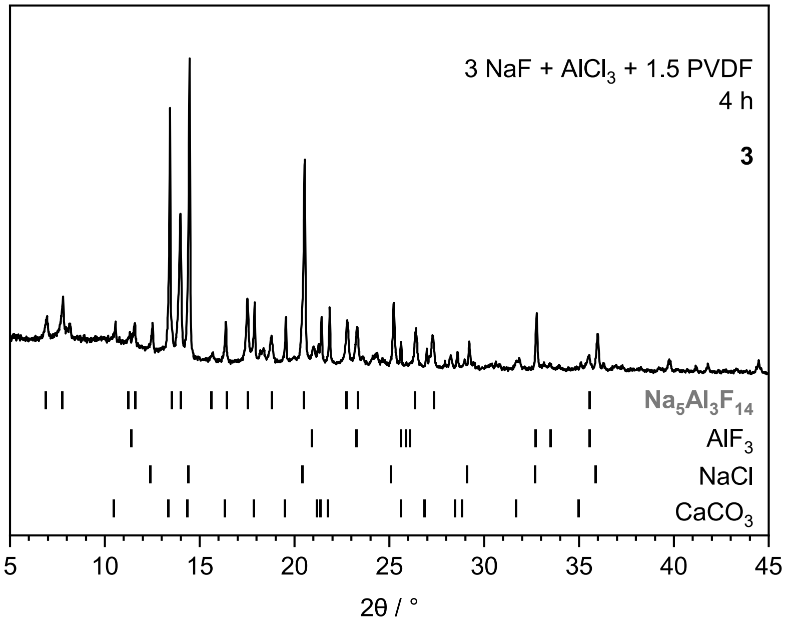

SI Figure 8. PXRD diffractogram (Mo Kα source λ = 0.7107 Å) of milling approach entry 3 with $CaCO_3$ as internal standard.

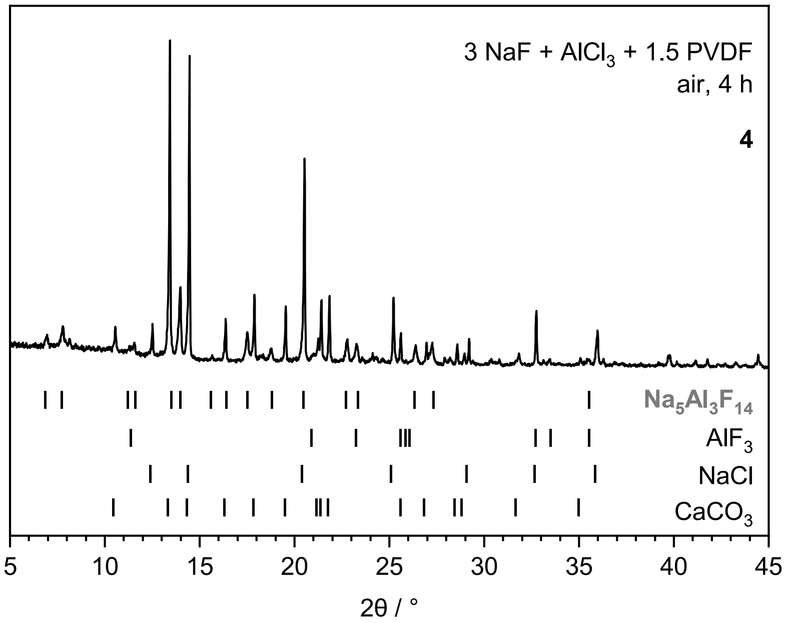

SI Figure 9. PXRD diffractogram (Mo Kα source λ = 0.7107 Å) of milling approach entry 4 with $CaCO_3$ as internal standard.

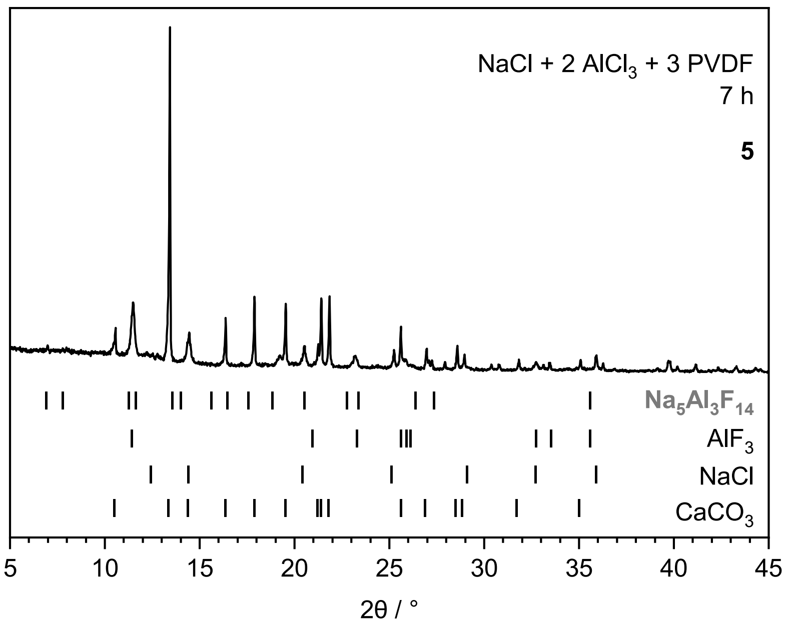

SI Figure 10. PXRD diffractogram (Mo Kα source λ = 0.7107 Å) of milling approach entry 5 with $CaCO_3$ as internal standard.

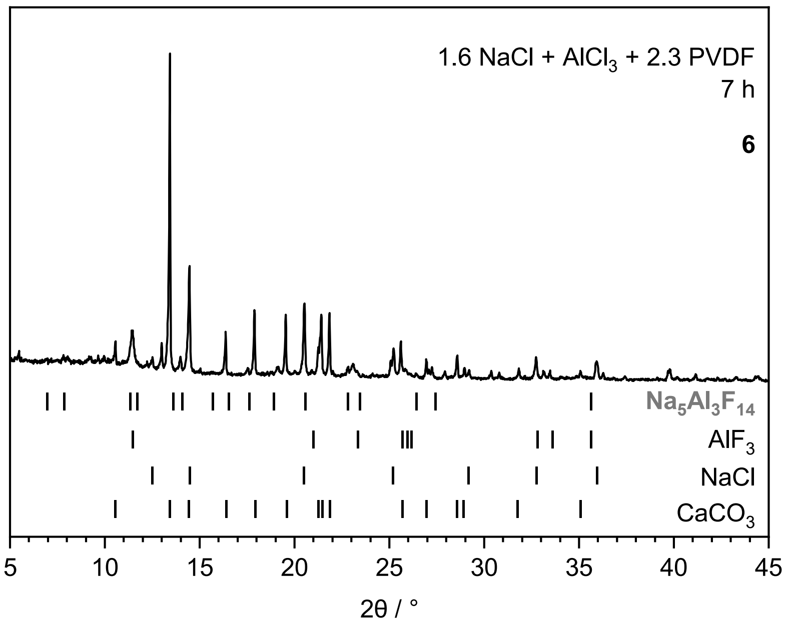

SI Figure 11. PXRD diffractogram (Mo Kα source λ = 0.7107 Å) of milling approach entry 6 with $CaCO_3$ as internal standard.

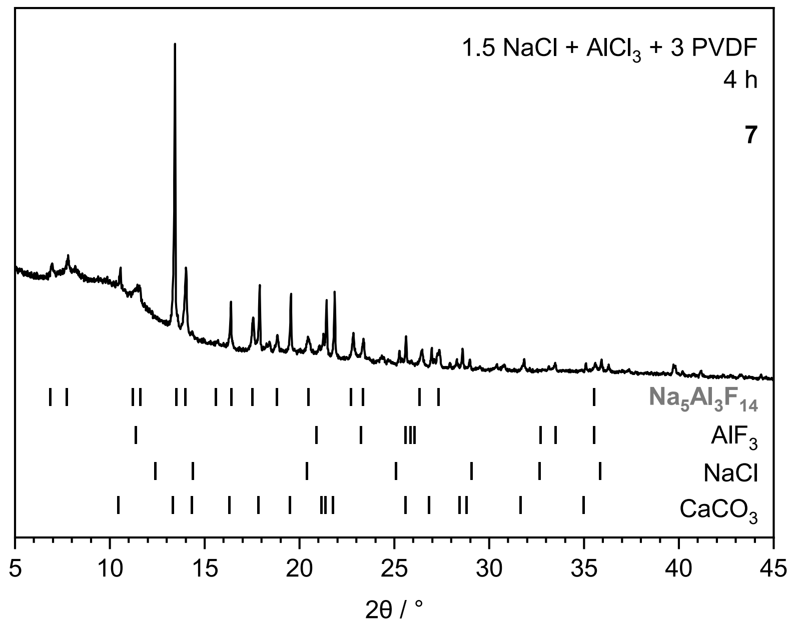

SI Figure 12. PXRD diffractogram (Mo Kα source λ = 0.7107 Å) of milling approach entry 7 with $CaCO_3$ as internal standard.

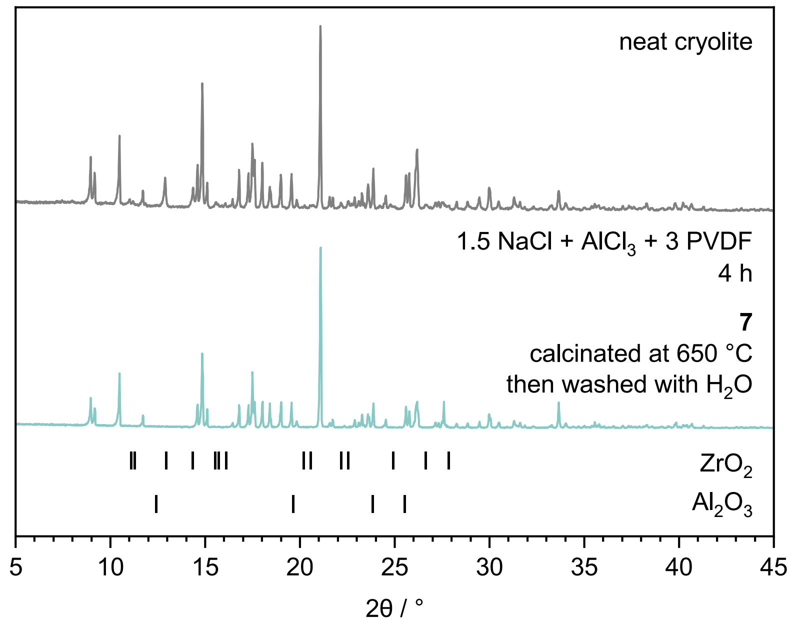

SI Figure 13. PXRD diffractogram (Mo Kα source λ = 0.7107 Å) of milling approach entry 7 in green (calcinated at 650 °C for 4 h and then washed with H_2O) and neat cryolite Na_3AlF_6 as reference in grey.

The ZrO_2 reflections in the PXRD pattern of SI Figure 13 are originated from abrasion of the ZrO_2 milling jar and balls. Rietveld refinement analysis of the powder mixture, calcinated in air at 650 °C for 4 h and then washed with H_2O reveals a content of 95 % cryolite, 1 % Al_2O_3 and 4 % ZrO_2

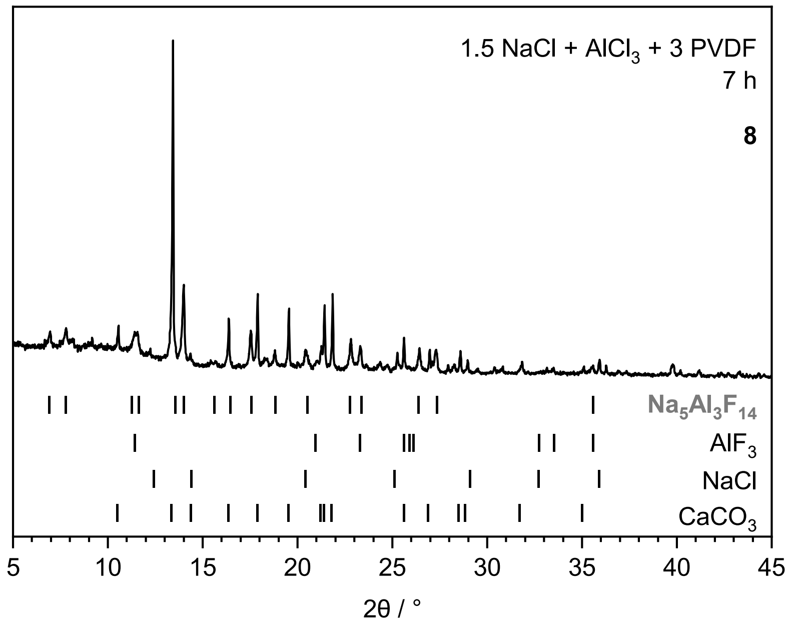

SI Figure 14. PXRD diffractogram (Mo Kα source λ = 0.7107 Å) of milling approach entry 8 with $CaCO_3$ as internal standard.

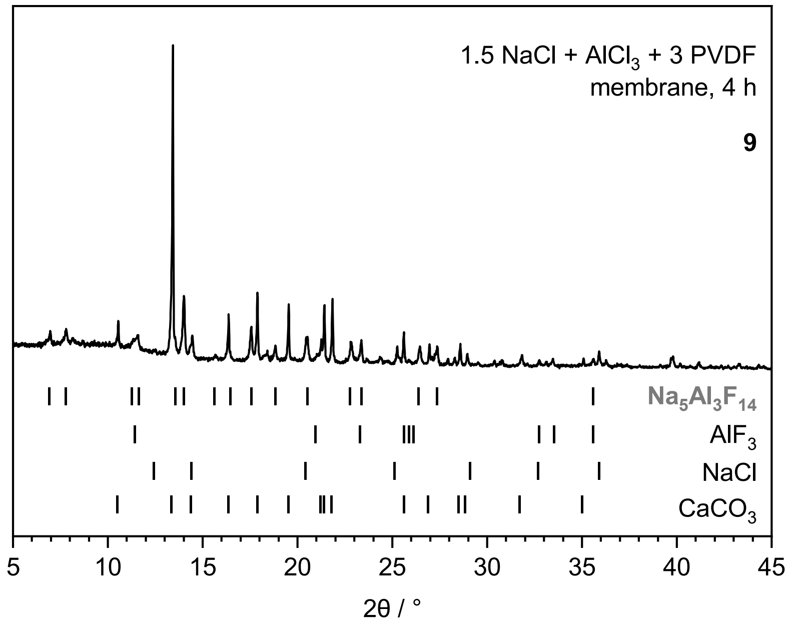

SI Figure 15. PXRD diffractogram (Mo Kα source λ = 0.7107 Å) of milling approach entry 9 using a PVDF membrane with $CaCO_3$ as internal standard.

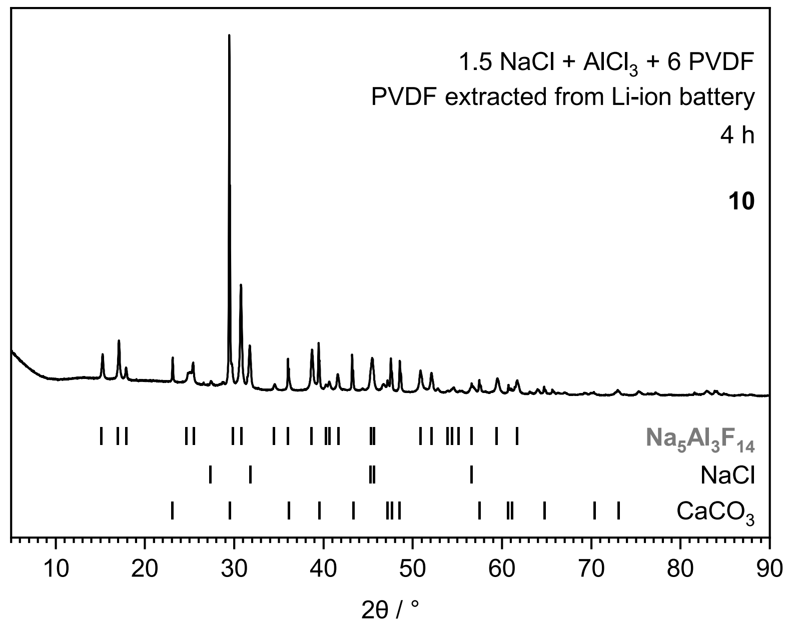

SI Figure 16. PXRD diffractogram (Cu Kα source λ = 1.5406 Å) of milling approach entry 10 (PVDF was extracted from a Li-ion battery) with $CaCO_3$ as internal standard.

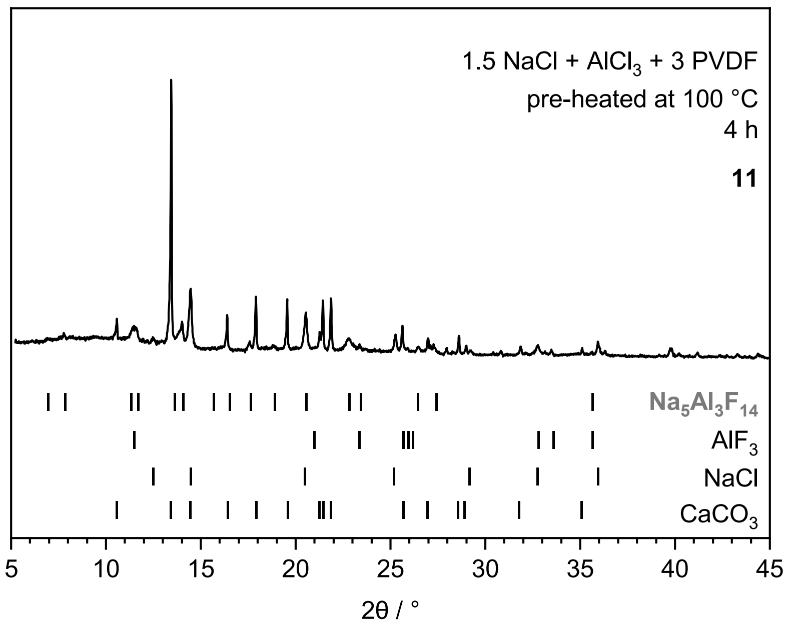

SI Figure 17. PXRD diffractogram (Mo Kα source λ = 0.7107 Å) of milling approach entry 11 (pre-heated jar at 100 °C) with $CaCO_3$ as internal standard.

Scanning / transmission electron microscopy

High-resolution transmission electron microscopy (HRTEM), high-angle annular dark-field scanning transmission electron microscopy (HAADF-STEM) and energy dispersive X-ray analysis (EDX) elemental mapping were carried out on a FEI Talos F200S scanning/transmission electron microscope (S/TEM) at an acceleration voltage of 200 kV. A dry TEM grid preparation was carried out. Therefore, TEM grids were carefully swiped across the powder samples. The excess of powder on the grids were removed by tapping lightly. The determination of the atomic fraction and atomic error by EDX analysis was conducted by averaging the data of five randomly selected spots on the particle.

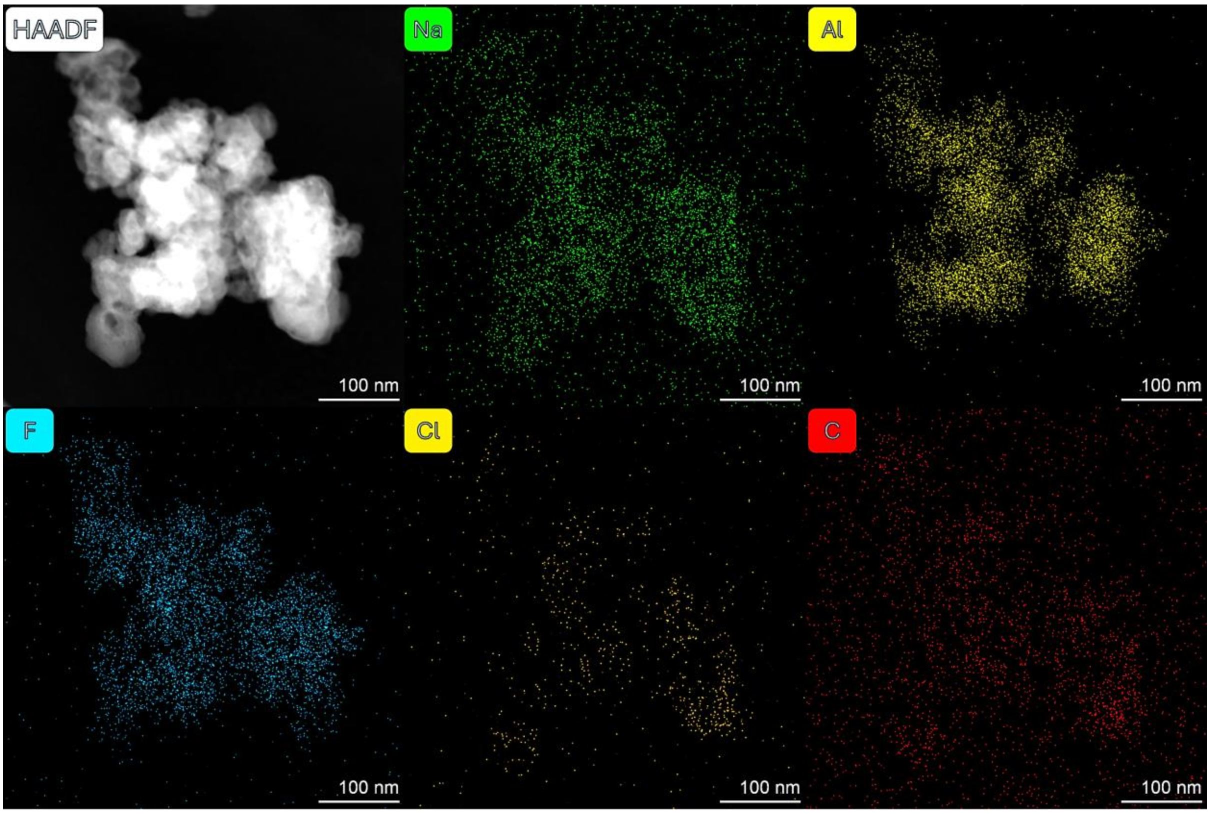

SI Figure 18. Elemental mapping of the sample obtained by the conditions shown in Entry 8 from SI Table 1 using STEM and EDX with a scale bar of 100 nm.

SI Table 2. EDX analysis of Entry 8.

Element	Atomic fraction / %	Atomic error / %
C	30.9	2.50
Cl	1.38	0.28
Na	23.75	3.93
Al	16.33	2.96
F	27.64	4.31

SI Table 2 shows the atomic composition by EDX of the powder mixture of Entry 8. Note that this analysis is not reliable. The powder mixture is a heterogeneous mixture containing sodium aluminium salt and residual starting compounds.

Solid-state MAS NMR spectroscopy

Solid-state MAS (magic angle spinning) nuclear magnetic resonance spectra were measured at a Bruker AVANCE 400 (B_0 = 9.4 T) spectrometer at room temperature. ^{1}H, ^{19}F and ^{27}Al MAS NMR spectra were measured on using 2.5 mm rotors at a rotation frequency of 25 or 20 kHz and a recycle delay of 5 s. ^{19}F-^{13}C-CP (cross polarisation) MAS NMR spectra were measured in 4 mm rotors at a rotation frequency of 10 kHz, a recycle delay of 3 s and a contact time of 10 ms. The proton decoupled ^{13}C MAS NMR spectra were performed at 10 kHz with a recycle delay of 20 ms. The chemical shifts were referenced to a $CFCl_3$ (δ = 0 ppm) standard for ^{19}F and a 1 M aqueous solution of $AlCl_3$ (δ = 0 ppm) for ^{27}Al. α-AlF_3 served as the external standard for both nuclei. Values of isotropic chemical shifts of ^{13}C are given with respect to $Si(CH_3)_4$ and are measured against adamantane as secondary standard. ^{23}Na MAS spectra were recorded with a recycle delay of 5 s. The spectra were referenced to 1 M aqueous solution of NaCl and NaF was used as a secondary standard.

SI Table 3. Isotropic chemical shift values (δ_{iso}) and quadrupolar frequency (ν_Q) for chiolite and cryolite of sample 8 (SI table 1) and calcinated sample 7 for 240 min at 650 °C in air.

Sample	Compound	^{27}Al			^{23}Na			^{19}F	
		Site	δ_{iso} ppm	ν_Q MHz	Site	δ_{iso} ppm	ν_Q MHz	Site	δ_{iso} ppm
**8**	Chiolite $Na_5Al_3F_{14}$	Al(1)	−18	0.7	Na(1)	−19	0.9	F(1)+F(3)	−190
		Al(2)	−9	1.1	Na(2)	−4	1.6	F(2)	−166
**7** calcinated	Cryolite Na_3AlF_6	$[AlF_6]$	0		Na(1)	2		F(1)+F(2) +F(3)	−190
					Na(2)	−8	0.7		

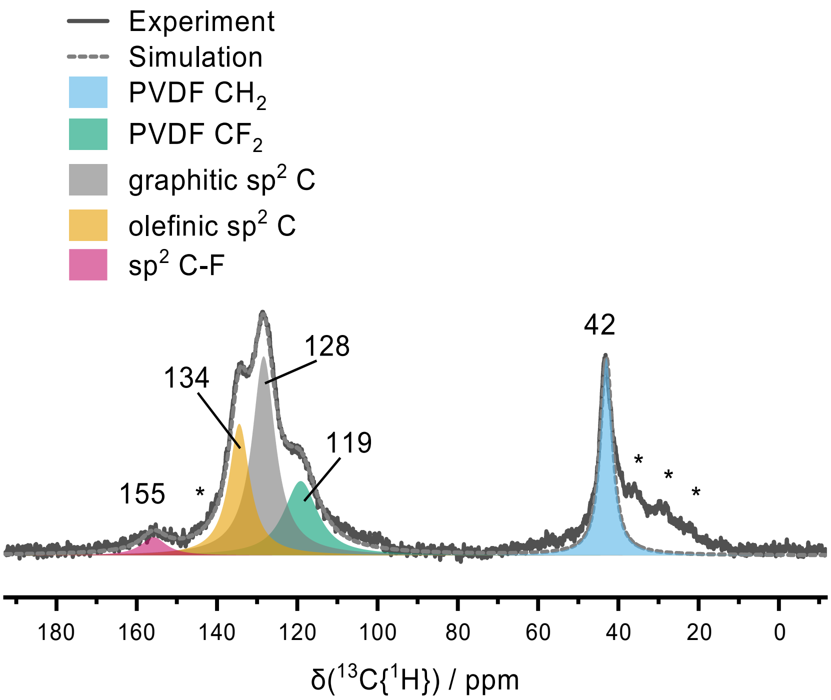

SI Figure 19. $^{13}C\{^1H\}$ MAS NMR spectrum ($\tilde{\nu}\tilde{\nu}_{rot}$ = 10 kHz) of the milling reaction entry 8 from SI Table 1. Asterisks (*) are representing spinning sidebands. Simulation of the spectra were conducted with DMFIT.[3]

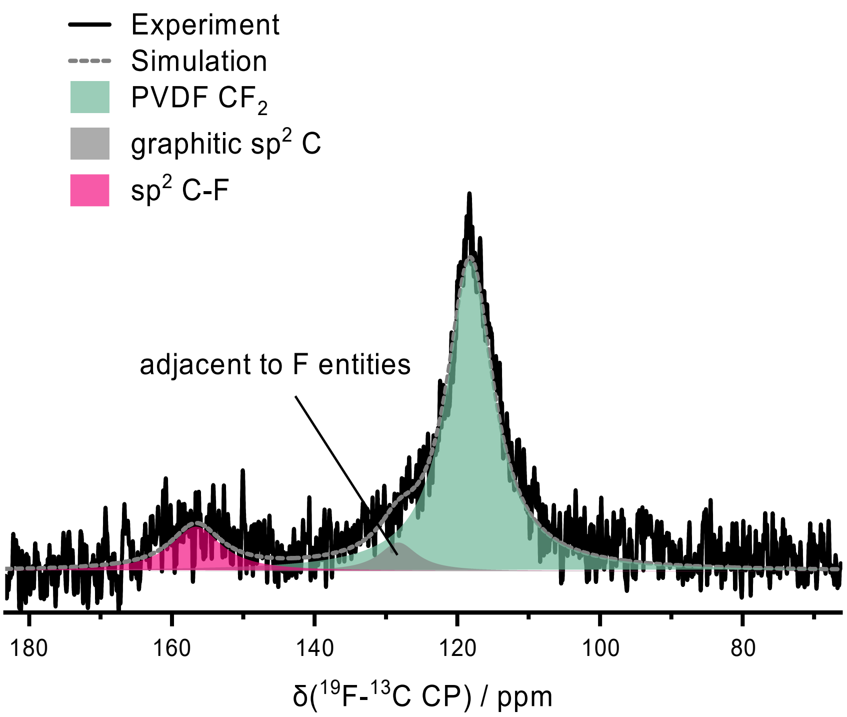

SI Figure 20. ^{19}F-^{13}C CP MAS NMR spectrum ($\tilde{\nu}\tilde{\nu}_{rot}$ = 10 kHz) of the milling reaction entry 8 from SI Table 1. Simulation of the spectra were conducted with DMFIT.[3]

The $^{13}C\{^{1}H\}$ MAS NMR spectrum of entry 8 from SI Table 1 (SI Figure 19) shows signals for the residual PVDF at 42 (CH_2) and 119 ppm (CF_2). At the typical chemical shift region for sp^2 C we observe two signals at 128 for graphitic sp^2 C and at 134 ppm for olefinic sp^2 C.[4-5] The signal for olefinic sp^2 C may rise from incomplete graphitisation of the polymer. At 155 ppm a signal for sp^2 C-F entities can be observed.

The ^{19}F-^{13}C CP MAS NMR spectrum of entry 8 from SI Table 1 (SI Figure 20) reveals three signals. The major signal is observed at 119 ppm for the residual PVDF (CF_2). The second minor signal is detected at 128 ppm for the graphitic sp^2 C atoms, which have weak interaction with adjacent fluorine atoms within the graphitic framework.[6] At 155 ppm a signal for sp^2 C–F can be assigned.[7]

Thermogravimetric analysis (TGA) and differential scanning calometry (DSC)

The TGA and DSC measurements were performed on a TGA/DSC 3+ from Mettler Toledo, Switzerland. Samples were weighed in in a glovebox and sealed with the A2 closing stamp. The closed corundum crucible was pinned in a N_2 or O_2 stream by the sample robot. The samples were heated from 25 to 800 °C at a rate of 10 K/min. Afterwards the samples were cooled down to 25 °C at the same rate.

PVDF was evaluated as a reference material via TGA and DSC under both oxidative O_2 and inert N_2 atmospheres (SI Figures 21 and 22). The polymer demonstrates significant thermal stability in both environments, remaining intact until approximately 400 °C. Beyond this threshold, a primary degradation phase was observed, which results in a mass reduction to roughly 40% for both samples. However, the final decomposition profiles diverged significantly based on the atmosphere. Under N_2, PVDF underwent incomplete degradation, retaining a residual mass of 35% even at 800°C. In contrast, the sample heated in O_2 reached complete degradation by 580°C. This oxidative breakdown was further characterised by the DSC thermogram, which recorded a distinct exothermic process between 450°C and 580°C, indicative of the combustion of the polymer chain.

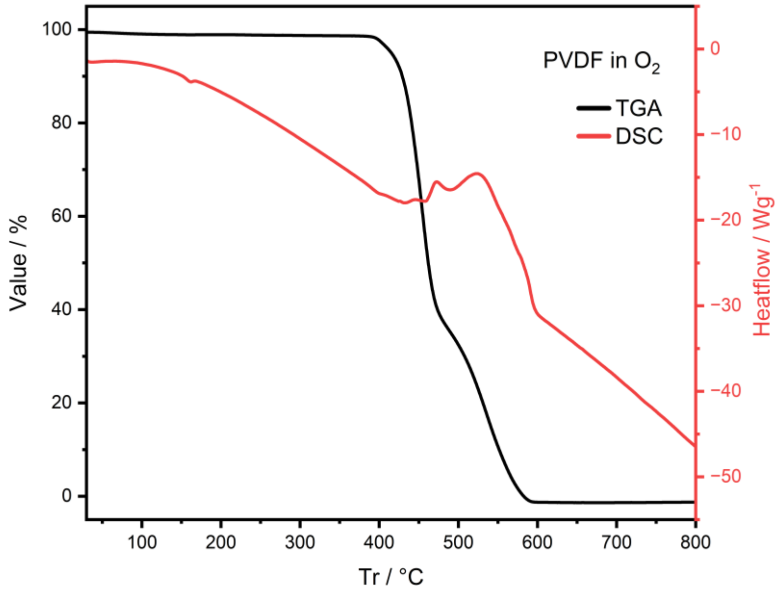

SI Figure 21. TGA and DSC diagram of PVDF heated in O_2.

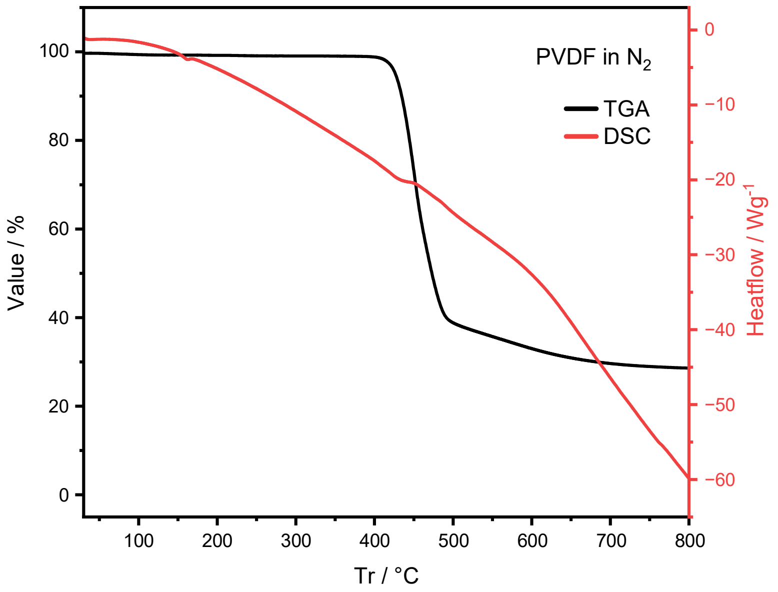

SI Figure 22. TGA and DSC diagram of PVDF heated in N_2.

The powder generated from the milling approach 7 was characterised using TGA and DSC under both oxidative O_2 and inert N_2 atmospheres. In an O_2 stream, the sample exhibited a gradual mass loss up to 450 °C, followed by a rapid thermal decomposition between 450 and 510 °C, where the residual mass dropped to 45 %. Beyond 510 °C, the degradation rate slowed, yielding a final mass of approximately 40 % at 800 °C. The corresponding DSC thermogram displays a prominent exothermic peak within the 450–510 °C range, which is attributed to the oxidative combustion of organic components within the milled powder. Conversely, under a N_2 atmosphere, the sample showed only a steady, minor mass loss across the entire temperature range without any abrupt transitions to a final mass of 60 % at 800 °C. Furthermore, the DSC trace

remained featureless, indicating an absence of significant thermodynamic events or phase transitions under inert conditions.

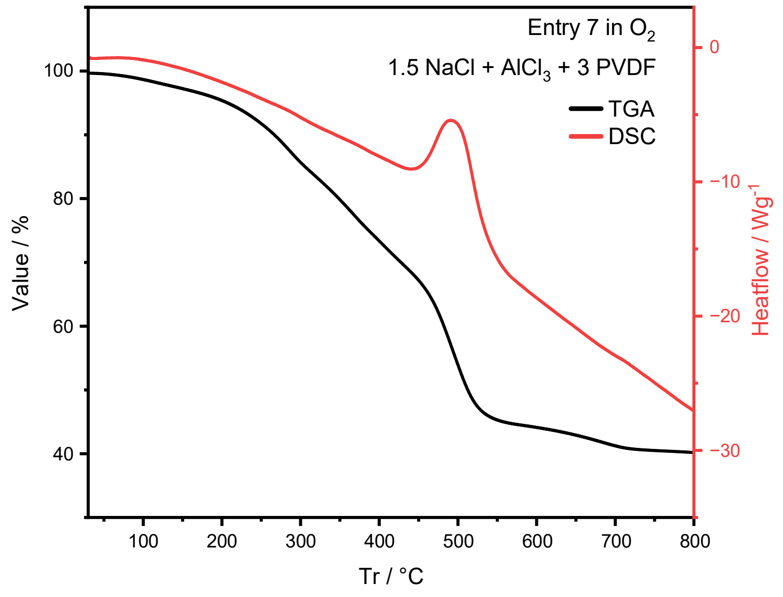

SI Figure 23. TGA and DSC diagram of entry 7 heated in O_2.

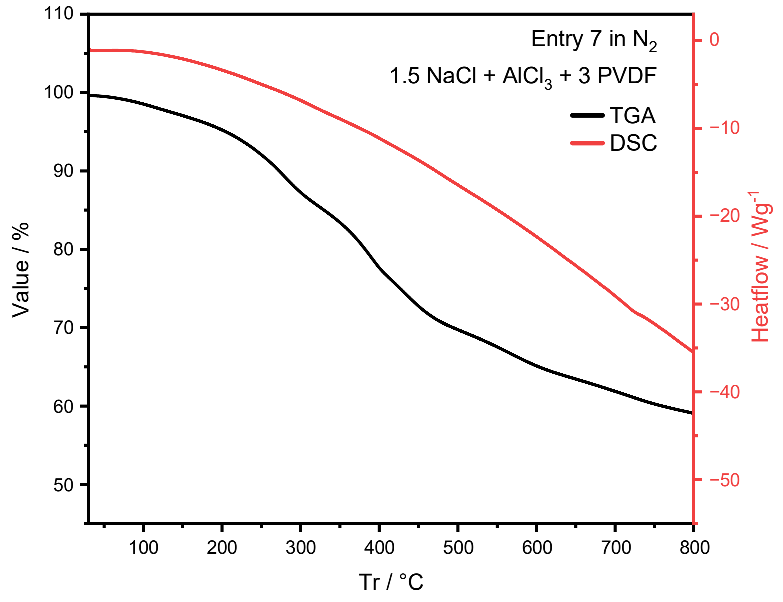

SI Figure 24. TGA and DSC diagram of entry 7 heated in N_2.

Laser-desorption ionisation (LDI) time of flight (TOF) Mass spectrometry

All experiments were performed on an Autoflex maX mass spectrometer from Bruker equipped with a 355 nm Nd-YAG laser and 2000 Hz repetition rate working in linear mode. Typically, 2000 shots recorded at 4 randomly chosen positions of the spot were

accumulated for one spectrum. The reproducibility of the results was checked by measuring multiple sample spots. Data recording, calibration and evaluation was performed using the instrument software and Origin. No matrices were used for the measurements.

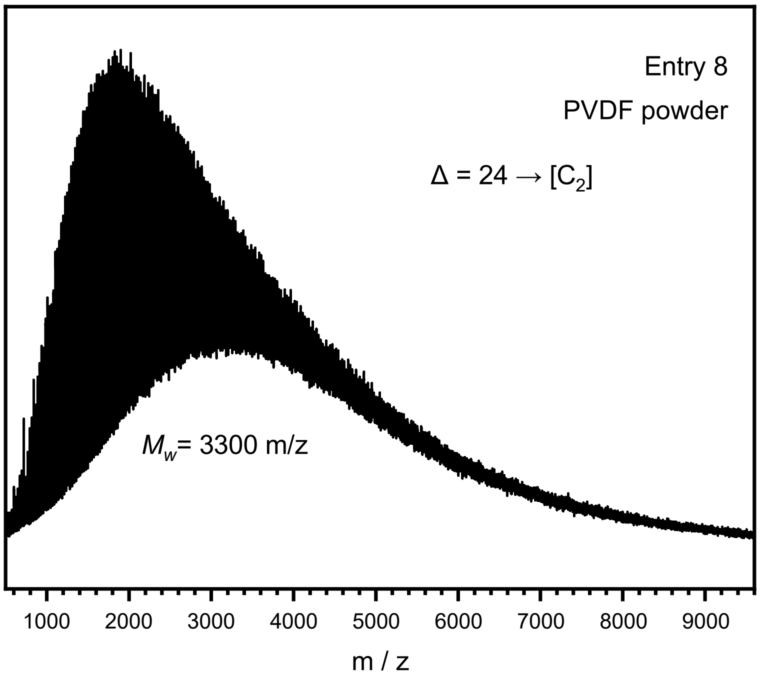

SI Figure 25. LDI-TOF data of the powder mixture of entry 8 from SI Table 1 using PVDF powder.

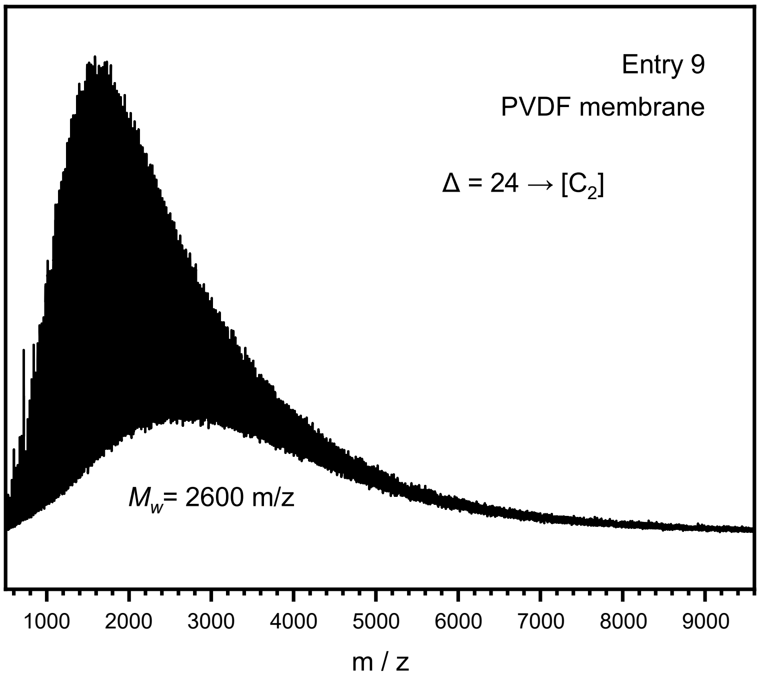

SI Figure 26. LDI-TOF data of the powder mixture of entry 9 from SI Table 1 using PVDF membrane.

Infrared spectroscopy

The IR spectra were recorded in an MBraun glovebox filled with argon at a Bruker Alpha II spectrometer with a diamond ATR (attenuated total reflectance) measuring unit (Pyroelectric DTGS detector).

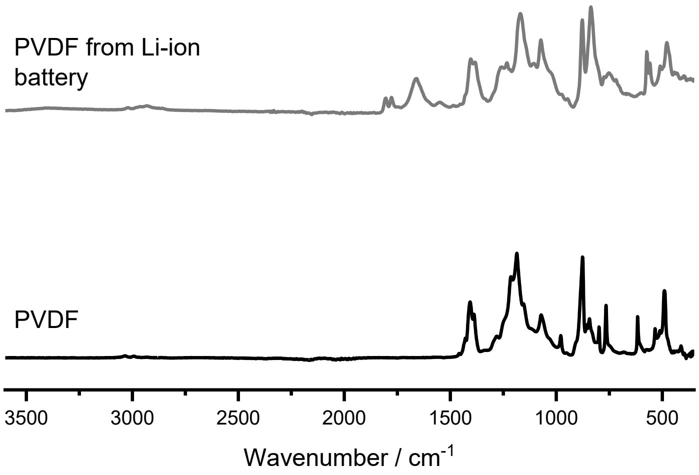

SI Figure 27. ATR IR spectra of PVDF from a Li-ion battery and neat PVDF.

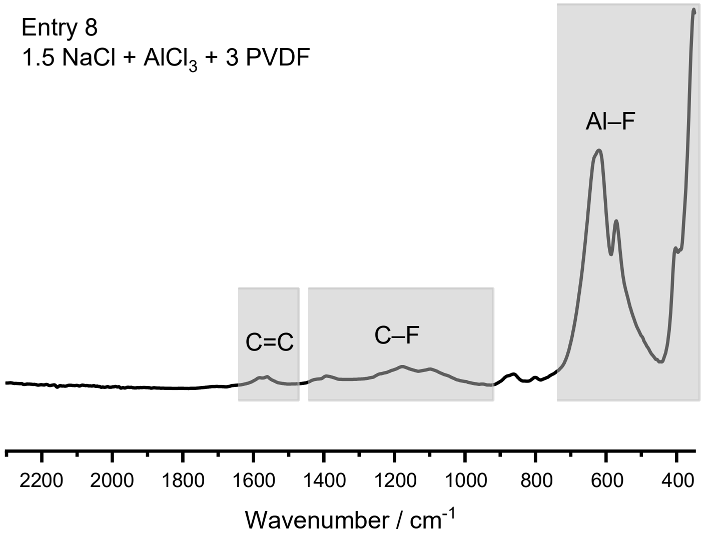

SI Figure 28. ATR IR spectrum of Entry 8 from SI Table 1.

Raman spectroscopy

Raman spectra were collected using a JASCO NRS-4100 Raman spectrometer. The spectrometer was equipped with a 1650 x 256 CCD detector (Andor; air/Peltier-cooled, operating temperature: −61 °C), a 900 L/mm grating, a diode laser with excitation of 532 nm, and a 100X (NA 0.90) objective. The laser power was maintained at 5.6 mW. Each Raman spectrum was recorded with an exposure time of 2 s and 3 accumulations.

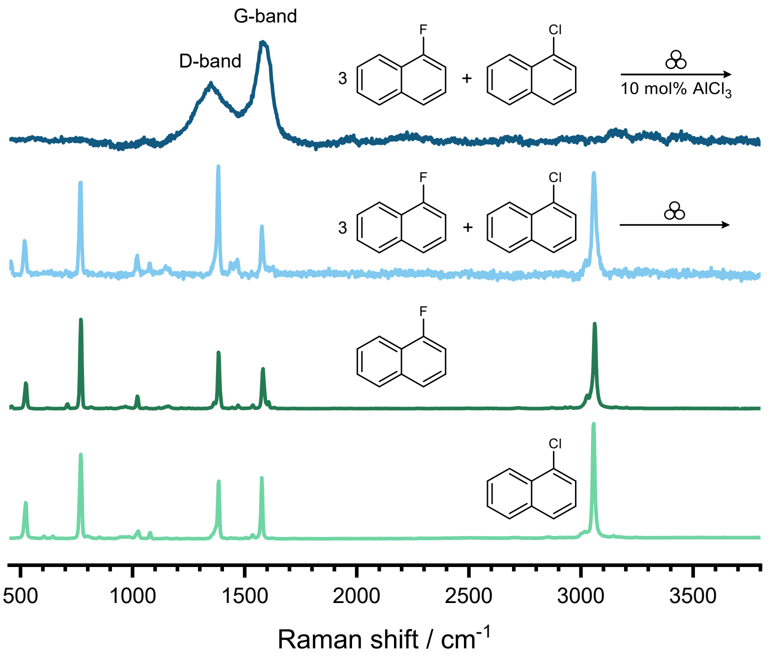

SI Figure 29. Raman spectra of neat 2-chloronapthalene, 2-fluoronapthalene, milling reaction of both naphthalene derivates with and without $AlCl_3$ acquired at 532 nm.

Milling 2-chloronaphthalene and 2-fluoronaphthalene together did not lead to the formation of graphitic entities. However, when a catalytic amount of $AlCl_3$ was added, a Scholl reaction takes place, converting the aryl rings into a graphitic material. This conversion is confirmed by the presence of G- and D-bands in the Raman spectrum as shown in SI Figure 15.

X-ray photoelectron spectroscopy

XPS analyses were conducted using the ULVAC-PHI “Quantes” spectrometer (Chanhassen, USA) with an Al Kα source (E = 1486.6 eV). The X-ray beam spot size

was adjusted to 100 µm for these experiments. Photoelectrons were gathered at a 45 ° emission angle. The Al Kα source was positioned with its X-ray beam perpendicular to the sample surface. The vacuum within the sample chamber was maintained below 10^{-6} Pa throughout the experiments. Analyses were conducted on three distinct areas of each sample, utilizing low energy electrons and Ar^{+} ions for charge neutralization. For the quantitative analysis, the survey spectra were used, which were measured with a pass energy of 280 eV and a step size of 1 eV. The binding energy (BE) scale was calibrated according to a PHI procedure that uses binding energy data from ISO 15472.[8] The intensity was calibrated with the PHI MultiPaK software with a method based on an idea of Seah.[9]

The percentage composition (in at-%) was determined from the peak areas after subtracting Shirley backgrounds and using relative sensitivity factors provided by the manufacturer with MultiPaK. PHI MultiPak Software Version 9.9.2 was used for the quantification of the atomic concentration using the automatic peak indexing routine. For the peak fitting with the Unifit software, the PHI datasets had to be converted from SPE to NPL format. For the peak fitting, Unifit 2025 (Unifit-Software, Leipzig, Germany) was employed using a sum of Gaussian-Lorentzian curves and a modified Tougaard background.[10]

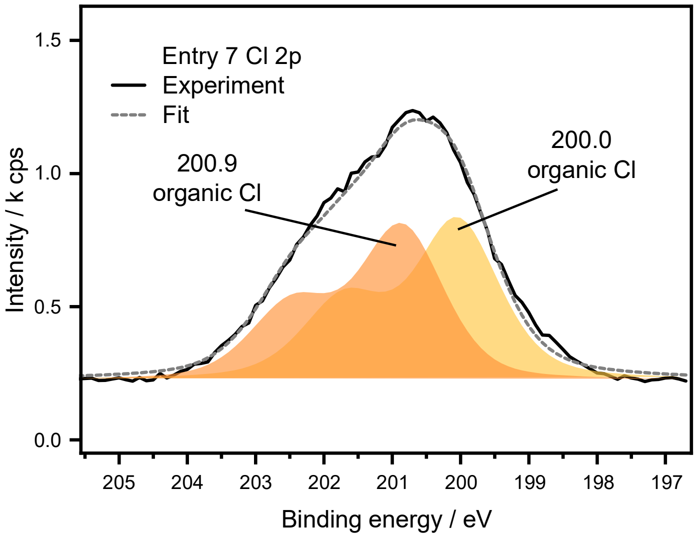

SI Figure 30. Cl 2p high resolution XPS spectrum of the milling sample 7 from SI table1.

The Cl 2p XPS spectrum of sample 7 shows two peaks at 200.0 and 200.9 eV indicative for organic chlorine entities.

SI Table 4. XPS quantification of the powder mixture Entry 7 from SI Table 1.

Element	Atomic fraction / %	Atomic error / %
Al	4.5	2.9
C	47.7	2.3
Cl	5.3	0.6
F	30.9	1.7
Na	8.8	0.7
O	0.8	0.4

SI Table 5. C 1s XPS analysis of Entry 7 from SI Table 1.

C 1s	Peak name	Peak height / cps	Position / eV	FWHM / eV	Rel. Area / %
**Entry 7**	C–C, C–H	2404.2	284.9	1.73	53.0
	CH_2, CHCl	1306.7	286.2	1.40	25.8
	CCl	484.7	287.3	1.41	9.6
	CCl_2	187.6	288.4	1.40	3.7
	CHF	174.6	289.6	1.40	3.4
	CF_2	227.6	290.8	1.40	4.5

SI Table 6. F 1s XPS analysis of Entry 7 from SI Table 1.

F 1s	Peak name	Peak height / cps	Position / eV	FWHM / eV	Rel. Area / %
**Entry 7**	Al–F	3860.7	687.6	2.5	89.2
	CF_x [a]	468.1	689.1	2.5	10.8

[a] x = 1, 2

SI Table 7. Al 2p XPS analysis of Entry 7 from SI Table 1.

Al 2p	Peak name	Peak height / cps	Position / eV	FWHM / eV	Rel. Area / %
**Entry 7**	Al–F	233.6	77.6	2.4	100.0

SI Table 8. Na 1s XPS analysis of Entry 7 from SI Table 1.

Na 1s	Peak name	Peak height / cps	Position / eV	FWHM / eV	Rel. Area / %
**Entry 7**	$Na_5Al_3F_{14}$[a]	1687.1	1073.6	2.4	100.0

[a] Note that no Na 1s XPS data for $Na_5Al_3F_{14}$ are published to date.

SI Table 9. Cl 2p XPS analysis of Entry 7 from SI Table 1.

Cl 2p	Peak name	Peak height / cps	Position / eV	FWHM / eV	Rel. Area / %
**Entry 7**	organic Cl	684.7	200.0	1.6	50.9
	organic Cl	681.4	200.9	1.6	49.1

Analytics for the gaseous content

After milling the substrates, the gaseous content from the jars were vacuum transferred into a JYoung NMR tube filled with 0.6 mL C_6D_6 at −196 °C. The solutions were used for NMR spectroscopic investigations.

Optical feedback cavity enhanced absorption spectroscopy (OFCEAS)

Low pressure sampling optical feedback cavity enhanced absorption spectroscopy (OFCEAS) was conducted at a ProCeas AP2E analyser from Durag Group. The analyser had a 20 km laser path length and was operated at a reduced pressure of

0.1 bar in the inlet. The measuring cell was maintained at a constant temperature of 40 °C. A 100 µm nozzle was used to achieve a sampling flow rate of 250 mL_n/min with H_2 as the matrix gas. The pipes and measuring cell were coated with SilcoNert® to minimise adsorption. An analyte specific laser was used to detect CO, CO_2, HCl and CH_4. The milling jars after reaction were directly connected to the OFCEAS device for the measurement.

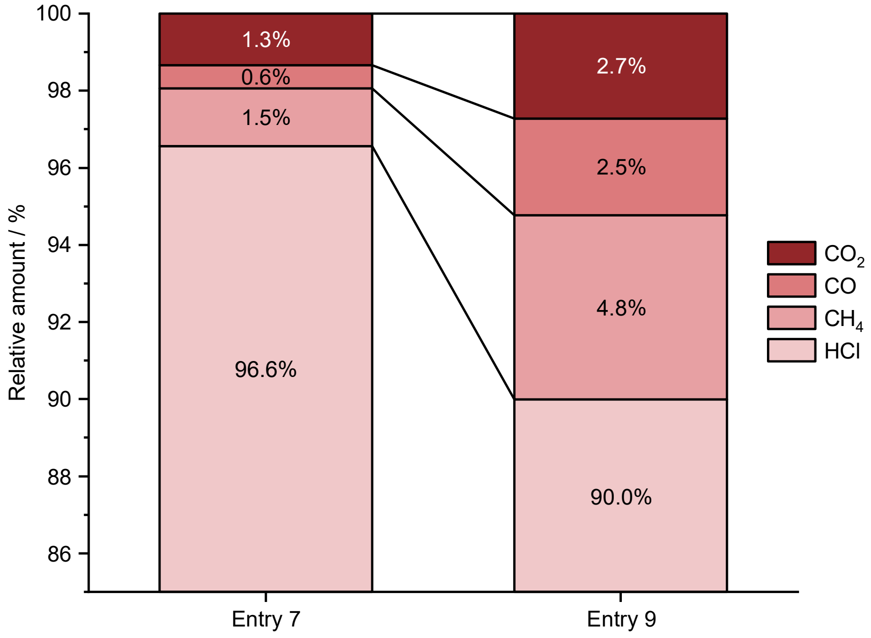

SI Figure 31. OCFEAS analysis of the gaseous content of the milling approach from Entry 7 and 8 from SI Table 1.

OFCEAS analysis (SI Figure 31) reveal the generation of mainly HCl and small amounts of CH_4. The generation of CO_2 and CO could be caused by the ingress of air due to imperfect sealing of the jars.

Liquid NMR spectroscopy

Liquid NMR spectra were recorded at Bruker DPX 300, Bruker AVANCE II 300, or Bruker AVANCE II 500 spectrometers at room temperature using tetramethyl silane (TMS) as external standard. ^{1}H NMR chemical shifts δ were referenced to residual C_6D_5H (δ = 7.16 ppm). ^{19}F NMR spectra were calibrated externally to $CFCl_3$ (δ=0 ppm).

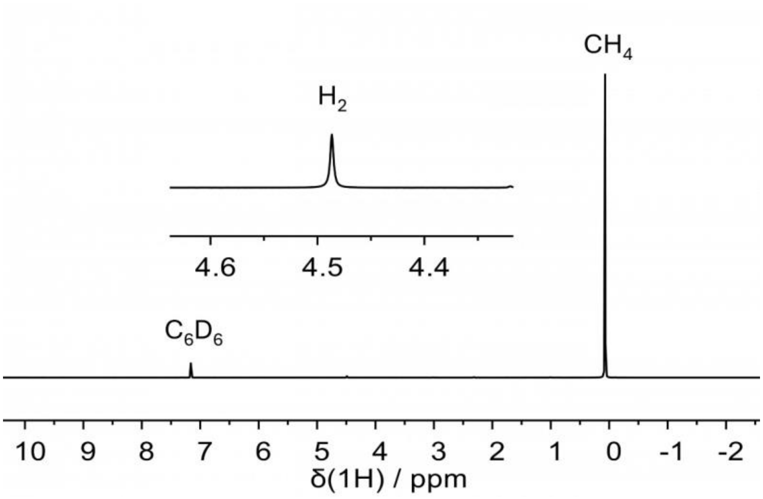

SI Figure 32. ^{1}H NMR (300 MHz, C_6D_6) spectrum of the gaseous content of Entry 7 from SI Table 1.

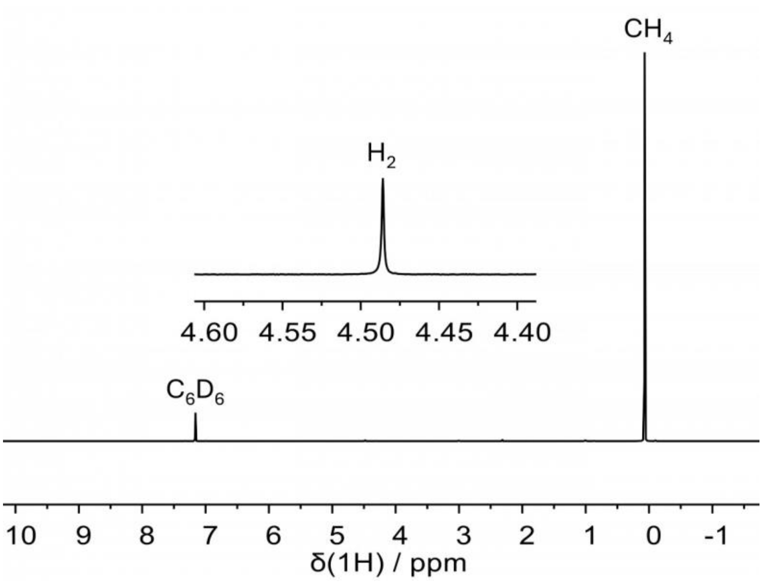

SI Figure 33. ^{1}H NMR (300 MHz, C_6D_6) spectrum of the gaseous content of Entry 9 from SI Table 1 using a PVDF membrane.

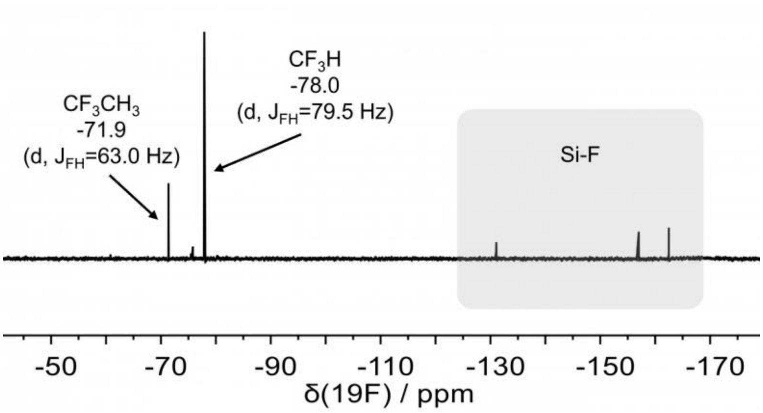

SI Figure 34. ^{19}F NMR (284 MHz, C_6D_6) spectrum of the gaseous content of Entry 7 from SI Table 1. HF may have reacted with the glass from the JYoung NMR tube to form Si–F compounds.

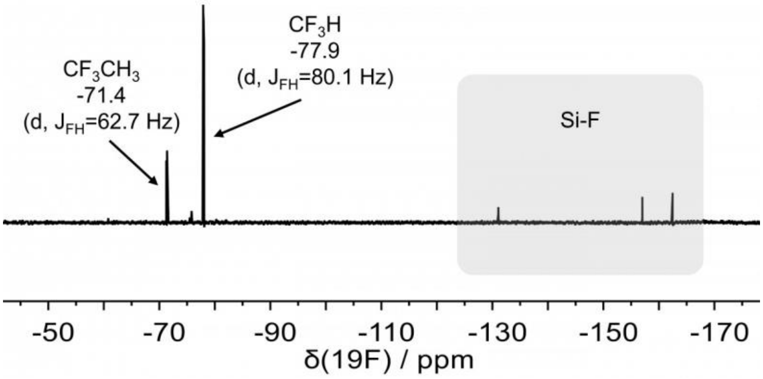

SI Figure 35. ^{19}F NMR (284 MHz, C_6D_6) spectrum of the gaseous content of Entry 9 from SI Table 1 using a PVDF membrane. HF may have reacted with the glass from the JYoung NMR tube to form Si–F compounds.

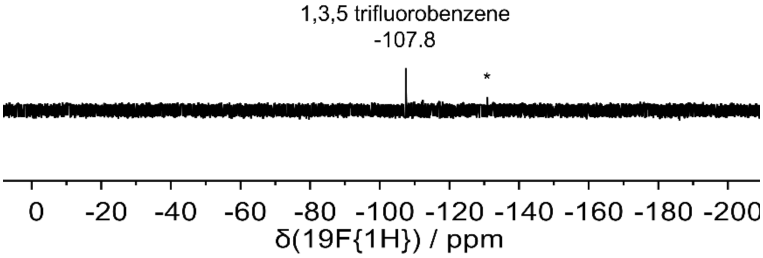

SI Figure 36. $^{19}F\{^1H\}$ NMR (284 MHz, C_6D_6) spectrum of the gaseous content from a milling mixture consisting of NaCl, $AlCl_3$, PVDF and AIBN after 30 min at 800 rpm. (*) HF may have reacted with the glass from the JYoung NMR tube to form Si–F compounds.

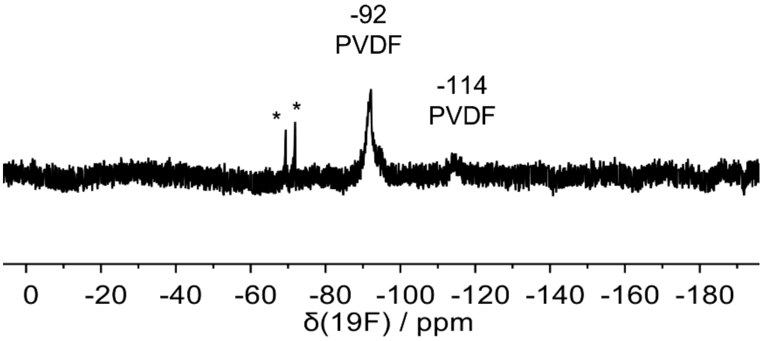

SI Figure 37. ^{19}F NMR (284 MHz, DMSO-d_6) spectrum of PVDF extracted from a Li-ion battery. The signal at −114 ppm is assigned to $-(CH_2CF_2CF_2CH_2)_n-$ motifs within PVDF. Asterisks (*) are assigned to impurities.

References

[1] N. Döbelin, R. Archer, V. Tu, "A free and open-source solution for Rietveld refinement of XRD data from the CheMin instrument onboard the Mars rover Curiosity", Planetary and Space Science **2022**, 224, 105596.

[2] H. Wang, M. Rehwoldt, D. J. Kline, T. Wu, P. Wang, M. R. Zachariah, "Comparison study of the ignition and combustion characteristics of directly-written Al/PVDF, Al/Viton and Al/THV composites", Combust. Flame **2019**, 201, 181-186.

[3] D. Massiot, F. Fayon, M. Capron, I. King, S. Le Calvé, B. Alonso, J.-O. Durand, B. Bujoli, Z. Gan, G. Hoatson, "Modelling one- and two-dimensional solid-state NMR spectra", Magn. Reson. Chem. **2002**, 40, 70-76.

[4] F. A. L. de Souza, A. R. Ambrozio, E. S. Souza, D. F. Cipriano, W. L. Scopel, J. C. C. Freitas, "NMR Spectral Parameters in Graphene, Graphite, and Related Materials: Ab Initio Calculations and Experimental Results", J. Phys. Chem. C **2016**, 120, 27707-27716.

[5] R. A. Friedel, H. L. Retcofsky, "Carbon-13 Nuclear Magnetic Resonance Spectra of Olefins and Other Hydrocarbons", J. Am. Chem. Soc. **1963**, 85, 1300-1306.

[6] K. Guérin, J. P. Pinheiro, M. Dubois, Z. Fawal, F. Masin, R. Yazami, A. Hamwi, "Synthesis and Characterization of Highly Fluorinated Graphite Containing sp2 and sp3 Carbon", Chem. Mater. **2004**, 16, 1786-1792.

[7] R. S. Matthews, "19F NMR of five polyfluoronaphthalenes. Inter-ring FF coupling constants", Org. Magn. Reson. **1982**, 18, 226-230.

[8] I. 15472:, ISO Geneva, **2010**.

[9] M. P. Seah, "A system for the intensity calibration of electron spectrometers", J. Electron. Spectrosc. Relat. Phenom. **1995**, 71, 191-204.

[10] R. Hesse, R. Denecke, "Improved Tougaard background calculation by introduction of fittable parameters for the inelastic electron scattering cross-section in the peak fit of photoelectron spectra with UNIFIT 2011", Surf. Interface Anal. **2011**, 43, 1514-1526.
